# Current and emerging trends in techniques for plant pathogen detection

**DOI:** 10.3389/fpls.2023.1120968

**Published:** 2023-05-08

**Authors:** Marc Venbrux, Sam Crauwels, Hans Rediers

**Affiliations:** ^1^ Centre of Microbial and Plant Genetics, Laboratory for Process Microbial Ecology and Bioinspirational Management (PME&BIM), Department of Microbial and Molecular Systems (M2S), KU Leuven, Leuven, Belgium; ^2^ Leuven Plant Institute (LPI), KU Leuven, Leuven, Belgium

**Keywords:** plant pathogens, agriculture, pathogen detection, PCR-based detection, cultivation-based detection, biosensors, immunologica detection, sequencing-based detection

## Abstract

Plant pathogenic microorganisms cause substantial yield losses in several economically important crops, resulting in economic and social adversity. The spread of such plant pathogens and the emergence of new diseases is facilitated by human practices such as monoculture farming and global trade. Therefore, the early detection and identification of pathogens is of utmost importance to reduce the associated agricultural losses. In this review, techniques that are currently available to detect plant pathogens are discussed, including culture-based, PCR-based, sequencing-based, and immunology-based techniques. Their working principles are explained, followed by an overview of the main advantages and disadvantages, and examples of their use in plant pathogen detection. In addition to the more conventional and commonly used techniques, we also point to some recent evolutions in the field of plant pathogen detection. The potential use of point-of-care devices, including biosensors, have gained in popularity. These devices can provide fast analysis, are easy to use, and most importantly can be used for on-site diagnosis, allowing the farmers to take rapid disease management decisions.

## 1 Introduction

Plant pathogens currently pose a major threat towards the agricultural industry. Up to 40% of the yield of economically important crops is lost each year due to plant pathogens and pests (FAO, 2019; Savary et al., 2019; Baldi and La Porta, 2020). The losses associated with plant disease carry a high economic burden, with an estimated annual loss of $220 billion dollars (FAO, 2019). Next to the economic impact of plant pathogens, the socio-ecological impact cannot be underestimated, considering that the world population is set to increase to 9.7 billion by the year 2050, which goes hand in hand with an increased global food consumption (FAO, 2017). In the past, the increased food demand was met by an increase in agricultural land use. However, this comes at the expense of other spatial arrangements such as living space, industry, but maybe most importantly forestry (McDonald and Stukenbrock, 2016). Considering 50% of the habitable land is already used for agriculture, the best practice seems to increase the yields that can be achieved, a strategy referred to as agricultural intensification (McDonald and Stukenbrock, 2016; Baldi and La Porta, 2020). However, intensification of agriculture, for instance by the dense cultivation of large areas with monocultures, facilitates the rapid spread of host-specialized pathogens (McDonald and Stukenbrock, 2016; Savary et al., 2019; Baldi and La Porta, 2020). A list of the most important plant pathogenic viruses, bacteria and fungi/oomycetes are listed in **Table 1**. The impact of these pathogens is aggravated by the increase of global trade, which accelerates the introduction of invasive pathogens and results in substantial crop damage and yield loss. Especially in underdeveloped countries these factors can have a devastating economic and social impact (Vurro et al., 2010; De Boer and López, 2012; Baldi and La Porta, 2020). Therefore, it is of huge importance to reduce the losses associated with plant disease. More importantly, these losses need to be reduced in a sustainable manner. The European Commission launched the Green Deal in 2019, an incentive to combat climate change and make industry and agriculture more sustainable (EU, 2019). One approach to contribute to the goals of the Green Deal consists of the effective implementation of integrated pest management (IPM), in order to reduce the environmental impact of conventional (usually chemical) disease management strategies. IPM revolves around the careful consideration of all plant protection methods available with the main aim to reduce chemical pesticides to levels which pose a minimal risk to either humans or the environment. To achieve this, biological control and cultivation techniques that can reduce disease or symptoms are encouraged. Additionally, if the use of a chemical pesticide cannot be avoided, its application should be highly specific for the target pathogen and should only be used when there is a real pathogen threat. The timely and accurate detection and identification of pathogens is therefore paramount for effective disease management strategies, as it allows for highly specific and localized remediation strategies, leading to reduced pesticide use and ultimately contributing to a more sustainable agriculture.

**Table 1:** Overview of the top 10 plant pathogens among viruses, bacteria, fungi and oomycetes (according to Scholthof et al., 2011; Dean et al., 2012; Mansfield et al., 2012; Kamoun et al., 2015).

Rank	Viruses	Bacteria	Fungi	Oomycetes	
**1**	Tobacco mosaic virus (TMV)	*Pseudomonas syringae* pathovars	*Magnaporthe oryzae*	*Phytophthora infestans*	
**2**	Tomato spotted wilt virus (TSWV)	*Ralstonia solanacearum*	*Botrytis cinerea*	*Hyaloperonospora arabidopsidis*
**3**	Tomato yellow leaf curl virus (TYLCV)	*Agrobacterium tumefaciens*	*Puccinia* spp.	*Phytophthora ramorum*	
**4**	Cucumber mosaic virus (CMV)	*Xanthomonas oryzae* pv. *oryzae*	*Fusarium graminearum*	*Phytophthora sojae*
**5**	Potato virus Y (PVY)	*Xanthomonas campestris* pathovars	*Fusarium oxysporum*	*Phytophthora capsici*	
**6**	Cauliflower mosaic virus (CaMV)	*Xanthomonas axonopodis* pv*. manihotis*	*Blumeria graminis*	*Plasmopara viticola*
**7**	African cassava mosaic virus (ACMV)	*Erwinia amylovora*	*Mycosphaerella graminicola*	*Phytophthora cinnamomi*	
**8**	Plum pox virus (PPV)	*Xylella fastidiosa*	*Colletotrichum* spp.	*Phytophthora parasitica*
**9**	Brome mosaic virus (BMV)	*Dickeya* (*dadantii* and *solani*)	*Ustilago maydis*	*Pythium ultimum*	
**10**	Potato virus X (PVX)	*Pectobacterium carotovorum* (and *P. atrosepticum*)	*Melampsora lini*	*Albugo candida*

**Figure 1:**
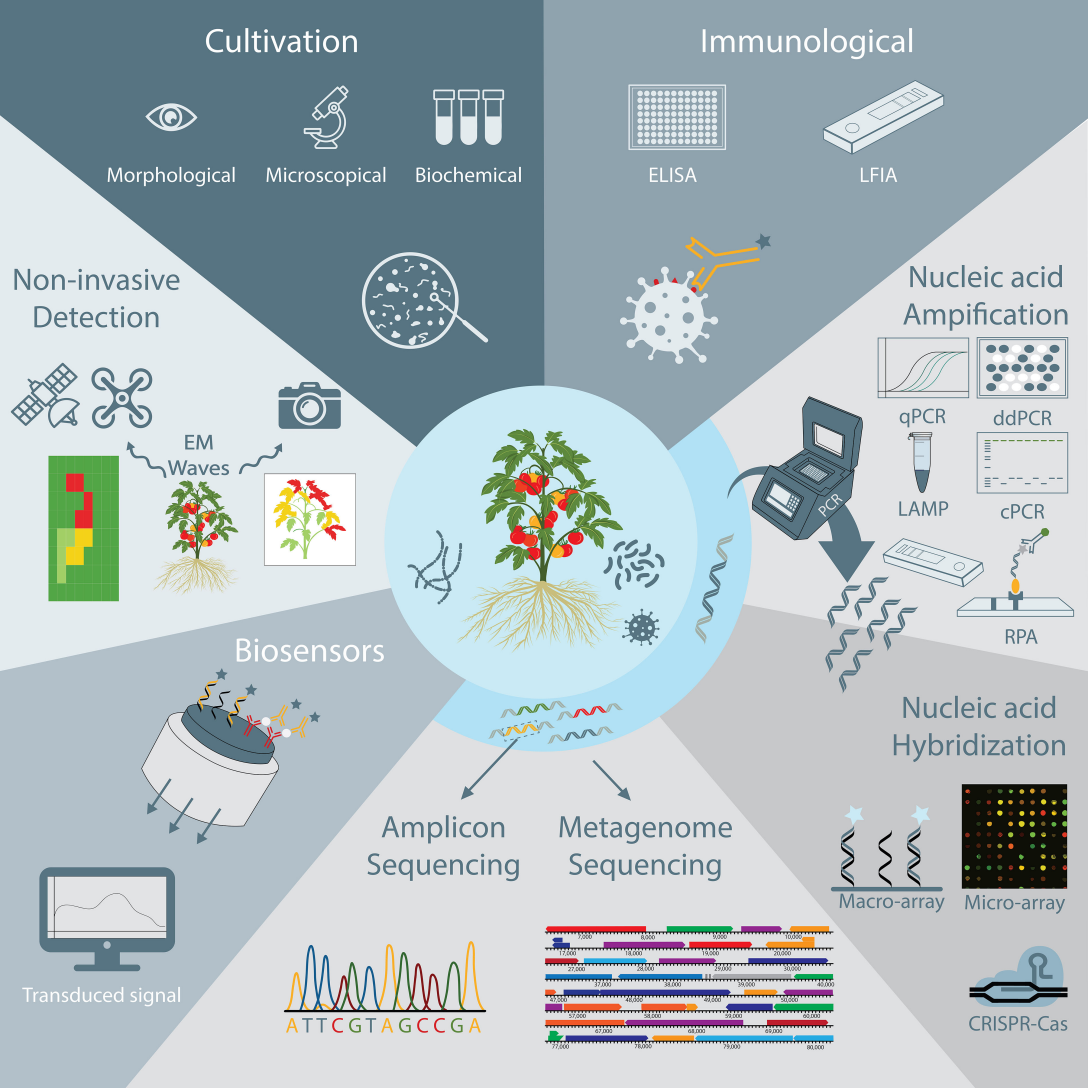
Schematic overview of plant pathogen detection techniques discussed in this review, including non-invasive monitoring, cultivation-based and immunological techniques, nucleic acid amplification and hybridization techniques, DNA sequencing techniques, and biosensors.

Ideally, a plant pathogen detection technique is specific, sensitive, accurate, reliable, fast, easy to use, cost-effective, and able to detect pathogens in complex matrices, such as soil samples or plant extracts. The main aim of this study is to give an overview of current and emerging trends in plant pathogen detection methods (**Figure 1**). This includes more conventional methods, such as cultivation-based, immunological and nucleic acid-based detection strategies, but also more advanced methods such as biosensors and high-throughput sequencing techniques. The methodology as well as the main advantages and disadvantages of currently available techniques are discussed, which should allow researchers and stakeholders to easily compare the different options that are available nowadays and select a method that is most suited for their specific use. In addition, we pointed to some relevant examples of how each technique was successfully used for detection of plant pathogens.

## 2 Non-invasive optical and spectral detection methods

**Figure 2:**
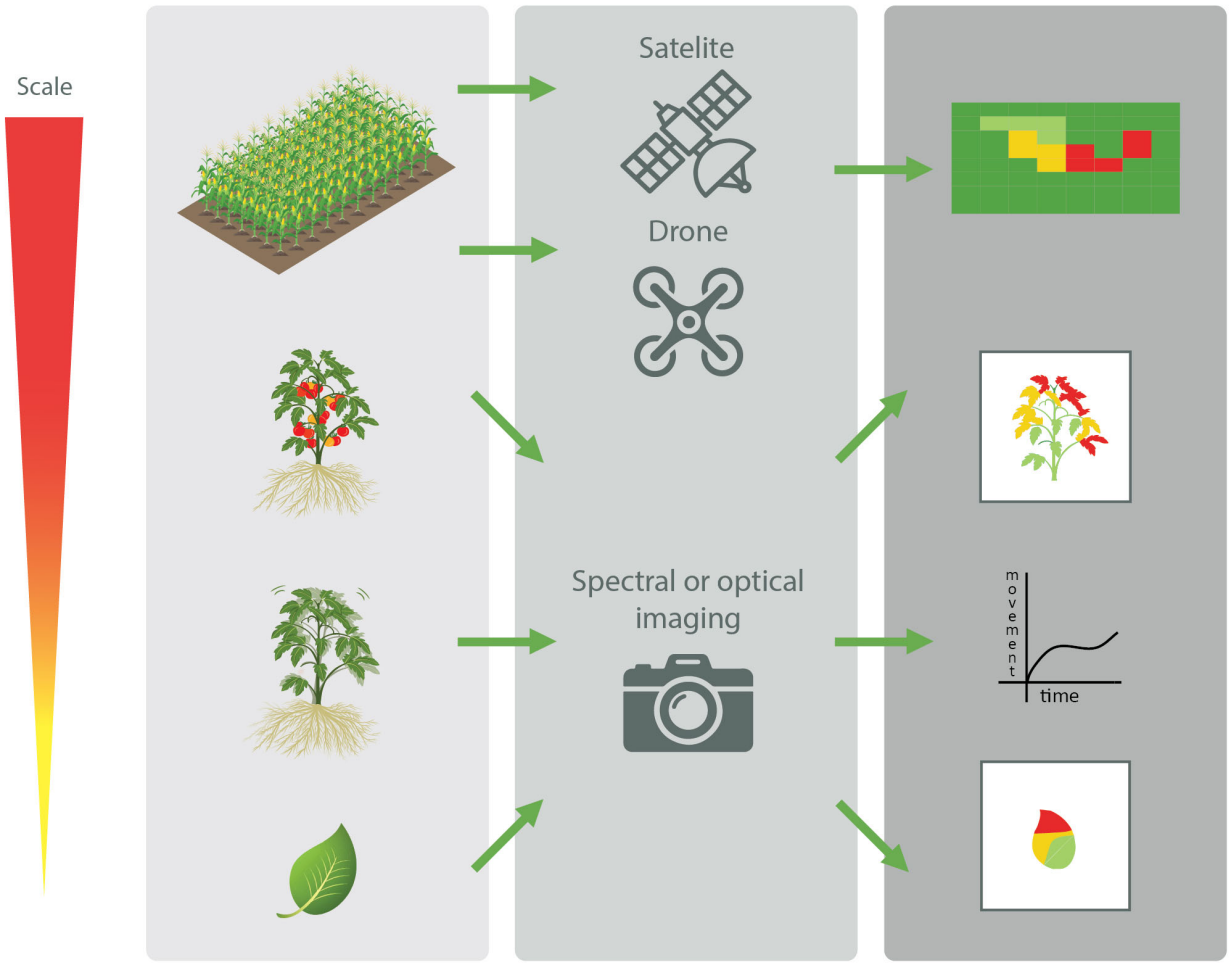
Schematic overview of the different scales at which non-invasive spectral and optical techniques of plant parts, plants, and entire fields can be used to detect biotic stresses in plants (Adapted from Singh et al., 2021).

A multitude of different tests are available to detect plant pathogens. Perhaps the easiest method is the visual detection of plant disease symptoms (Timmerman et al., 2014). However, visual detection doesn't allow to detect latent infections, in which the plant does not exhibit visual symptoms yet. In addition, efficient identification of the causative agent relies on the elaborate expertise of the observer and is prone to bias (Riley et al., 2002). Therefore, more objective techniques have been developed to detect plant pathogens. With the advent of digitalization, the use of imaging, and optical or spectral techniques in plant disease detection has seen a steady rise. Indeed, it has been shown that stressed or diseased plants produce a different spectral signature compared to that of healthy plants (Martinelli et al., 2015; Zubler and Yoon, 2020). For instance, upon biotic stresses, plants respond with changes in e.g., chlorophyl content and thermal radiation, and also show subtle plant movement changes. These changes will occur before disease symptoms such as wilting or leaf lesions are visible. Different advanced spectral methods are currently available to measure such changes in electromagnetic radiation emitted or reflected by the plants (Sankaran et al., 2010; Martinelli et al., 2015; Mahlein, 2016; Zubler and Yoon, 2020; Geldhof et al., 2021; Tanner et al., 2022). The spectral analysis can be applied at different scales, ranging from taking high resolution images from a single leaf, to utilizing drones taking spectral analyses of entire fields (**Figure 2**) (Singh et al., 2021). In addition, due to the ever-decreasing size, weight and cost of these sensors, their use can become more prevalent in the agricultural industry, with large automation potential for routine monitoring purposes. For example, the use of drones enables the routine monitoring of large cultivation areas, enabling to detect “hot-spots” of plants experiencing biotic stress. Taking this concept on a larger scale, high-resolution satellite imaging has shown its potential to detect biotic stress. For instance, Raza et al. (2020) recently reported that satellite imaging could be useful to detect sudden death syndrome in soybean, which is caused by *Fusarium virguliforme*. In that study, sudden death disease could be predicted even before visual symptoms occurred, and this with an accuracy of >75%.

The use of optical or spectral techniques has several advantages over other techniques: (i) detection can be done in real time by continuous monitoring of the crops; (ii) it can detect biotic stress; and (iii) it is a non-invasive detection method, which does not require any sample manipulations. However, while the data acquisition using optical sensors has become relatively easy, interpretation of the data to detect biotic stress in the plant can be highly complex, and requires development of specific algorithms, usually involving machine learning or neural networks (Zubler and Yoon, 2020). Moreover, while imaging techniques can detect biotic stress in the plant before visual symptoms appear, the technique still lacks discriminative capabilities to identify specific pathogens. It should therefore be combined with other more precise techniques to identify the causal pathogen to design a proper management strategy to tackle this particular pathogen. Nevertheless, by detecting the areas where the plants are exhibiting stress, easier, more targeted sampling can be deployed (Martinelli et al., 2015; Mahlein, 2016; Zubler and Yoon, 2020).

## 3 Cultivation-based methods

Cultivation-based methods are generally regarded as the gold standard for detection and identification of (plant) pathogens. The method relies on the cultivation and isolation of microorganisms on a selective or semi-selective growth medium, which allows the growth of the target pathogen, while inhibiting (or reducing) the growth of background microflora (Gopinath et al., 2014; Mancini et al., 2016; Ferone et al., 2020). Subsequently, the identity of the isolates that grow on the (semi-)selective growth medium needs to be confirmed by morphological, microscopical, biochemical, molecular, or immunological assays (Alvarez, 2004; Figdor and Gulabivala, 2011; Mandal et al., 2011; Gopinath et al., 2014; Mancini et al., 2016; Ferone et al., 2020). However, morphological and microscopical observations for identification of pathogens can be rather difficult and are often based on the interpretative skills and experience of the analyst (Rajapaksha et al., 2019). More objective methods consist of a series of biochemical and phenotypical tests to confirm the presumed identity of the clones cultivated on the (semi-) selective medium (Castro-Escarpulli et al., 2015). A wide variety of different biochemical or phenotypical tests exist, and the tests can be performed either manually or through the use of commercial kits and automated systems. Usually, commercial tests show higher reliability and sensitivity (Castro-Escarpulli et al., 2015). Examples include the use of the analytical profile index (API) systems and the Biolog™ microplates, which are based on the ability of the strain under investigation to utilize specific substrates (Smith et al., 1972; Geiss et al., 1985; Ieven et al., 1995; Shea et al., 2012; Ferone et al., 2020; API^®^, 2002). Alternative methods to identify microorganisms are matrix-assisted laser desorption/ionization in combination with time-of-flight analysis (MALDI-TOF) and fatty acid profiling. Although MALDI-TOF was developed for biomarker monitoring in general, it has proven its use for taxonomic identification of microorganisms as well (Ahmad et al., 2012; Chun et al., 2022). Alternatively, the fatty acid composition between microorganisms is variable and enables the generation of a unique lipid fingerprint for each organism. Analyzing the fatty acid profile allows taxonomic identification of the microorganism up to species level and has proven its use in plant pathogen identification as a cost-effective and rapid method (Yousef et al., 2012; Lacey et al., 2021). In addition, sequencing of dedicated genetic markers, also referred to as DNA barcoding, is also frequently used for taxonomic identification by comparing the DNA sequence of the genetic marker to previously identified sequences of known species. For taxonomic identification of bacteria, the 16S rRNA and *rpoB* genes are frequently used, while for fungi and oomycetes, the internal transcribed spacer (ITS) region is a common marker for identification (Antil et al., 2023).

The main advantages of cultivation-based methods are that they are simple and reliable, and do not require high-tech equipment. In addition, it allows to distinguish viable from non-viable organisms (Figdor and Gulabivala, 2011; Rajapaksha et al., 2019; Ferone et al., 2020; Li et al., 2020). With a performant selective medium, it also allows quantification of the target pathogen. The specificity is dependent on the battery of biochemical and phenotypical tests that are performed. The biochemical and phenotypical traits of the test strain are compared to those of reference strains to assess its identity (Castro-Escarpulli et al., 2015). Depending on the target microorganism and the matrix from which it is isolated, cultivation-based techniques can detect pathogens with a sensitivity of 10-10^4^ CFU/mL (López et al., 2003). Sensitivity of detection can be further improved if an enrichment step is included before plating, although in this case quantification is no longer possible. However, the cultivation-based method is far from ideal. Perhaps the biggest drawback of this methodology is the fact that not all microorganisms are culturable, which is generally referred to as the great plate count anomaly (Connon and Giovannoni, 2002). The method is also very time-consuming, and a conclusive result can take anywhere from days up to weeks, due to the time needed for the organisms to grow and for performing the series of assays to confirm their identity (Law et al., 2015; Ferone et al., 2020; Li et al., 2020). Lastly, cultivation on (semi-)selective media is not suitable to detect viral plant pathogens, due to their host-dependent nature. However, instead of cultivation on selective media, detection of viral pathogens can be achieved by detection of visible symptoms on indicator or prey plants. Such tests are also very time-consuming and can take weeks depending on the plant that is studied (Legrand, 2015; Mehetre et al., 2021).

Mainly due to its simplicity, cultivation-based techniques are still widely used. Regulatory agencies such as the European and Mediterranean Plant Protection Organization (EPPO) describe a set of standardized cultivation-based protocols for detection of a multitude of important plant pathogens (e.g., *Xanthomonas* spp., *Pseudomonas* spp., *Fusarium* spp., etc.). However, the EPPO procedures often advise to perform an additional DNA-based test, such as a conventional or real-time PCR assay, to confirm the identity of the pathogen (EPPO, 2022).

## 4 Immunological methods

Immunological or serological assays to detect plant pathogens comprise methods using specific antibodies. Microorganisms produce a wide variety of antigenic molecules that can be used for detection (Alvarez, 2004). The antibodies used in such assays bind to specific epitopes on these antigens (López et al., 2003; Fang and Ramasamy, 2015). Binding of the antibodies to the antigens can be detected by making use of specific antibodies that are conjugated with e.g., an enzyme, a fluorophore or a nanoparticle. In this way the presence of the pathogens can be determined in an indirect way (Fang and Ramasamy, 2015). Antibody-antigen interactions are very specific, and a wide variety of antibodies exist that target antigens of specific pathogenic microorganisms. In general, there are two types of antibodies that can be used: (i) polyclonal antibodies, which consists of a mixture of antibodies that have affinity for different epitopes of the antigen(s) of the target pathogen; or (ii) monoclonal antibodies, consisting of one type of antibody with specificity to a single epitope (Alvarez, 2004; Martinelli et al., 2015). As polyclonal antisera contain multiple antibodies that target different epitopes, there is a higher risk of cross-reactivity with other antigens, leading to false-positive results (Alvarez, 2004; Kumar et al., 2008; Martinelli et al., 2015). In this regard, hybridoma technology and phage display technology to produce monoclonal antibodies greatly revolutionized immunological assays (Kumar et al., 2008; Hammers and Stanley, 2014). The use of monoclonal antibodies considerably increases specificity and reproducibility, since there is no inter-batch variability as is the case with polyclonal antibodies (Alvarez, 2004). The drawback of using monoclonal antibodies is that they are generally more expensive and less sensitive than their polyclonal counterparts (Martinelli et al., 2015; Ascoli and Aggeler, 2018).

In theory, immunological assays are applicable to all plant pathogens that express antigenic molecules, and as such can be used for detection of bacterial, fungal, and viral pathogens (Alvarez, 2004; Mancini et al., 2016; Mehetre et al., 2021). Immunological assays do not always require the isolation of the pathogen, which simplifies their use, but this comes with a limited sensitivity (Mancini et al., 2016). However, in many cases the sensitivity of immunological assays is considerably increased by including a sample pretreatment step such as a heat treatment or lysozyme treatment (Jones et al., 1997; Lima et al., 2014). Additional strategies that result in a higher sensitivity include the employment of a (pre-) enrichment step, where the sample is incubated in a selective medium to increase the level of target pathogen (Välimaa et al., 2015). Immunomagnetic separation (IMS), involving the use of magnetic beads that are coated with antibodies specific to the target, can be employed to capture and concentrate the target pathogen, further improving the sensitivity of immunological assays and removing possible contaminants (Kohn, 1999; Välimaa et al., 2015).

There is a wide range of immunological assays available to detect pathogens, most of them used in a clinical context, including chemiluminescent immunoassays, fluorescence immunoassays, fluorescence automated cell sorting, latex agglutination assays, western blot, etc. The most frequently used immunological techniques that are used for detection of plant pathogens are discussed below. For an overview of other immunological assays, we refer to (Atmar, 2014; Ahmed et al., 2020; Haselbeck et al., 2022).

### 4.1 Enzyme linked immunosorbent assay (ELISA)

**Figure 3:**
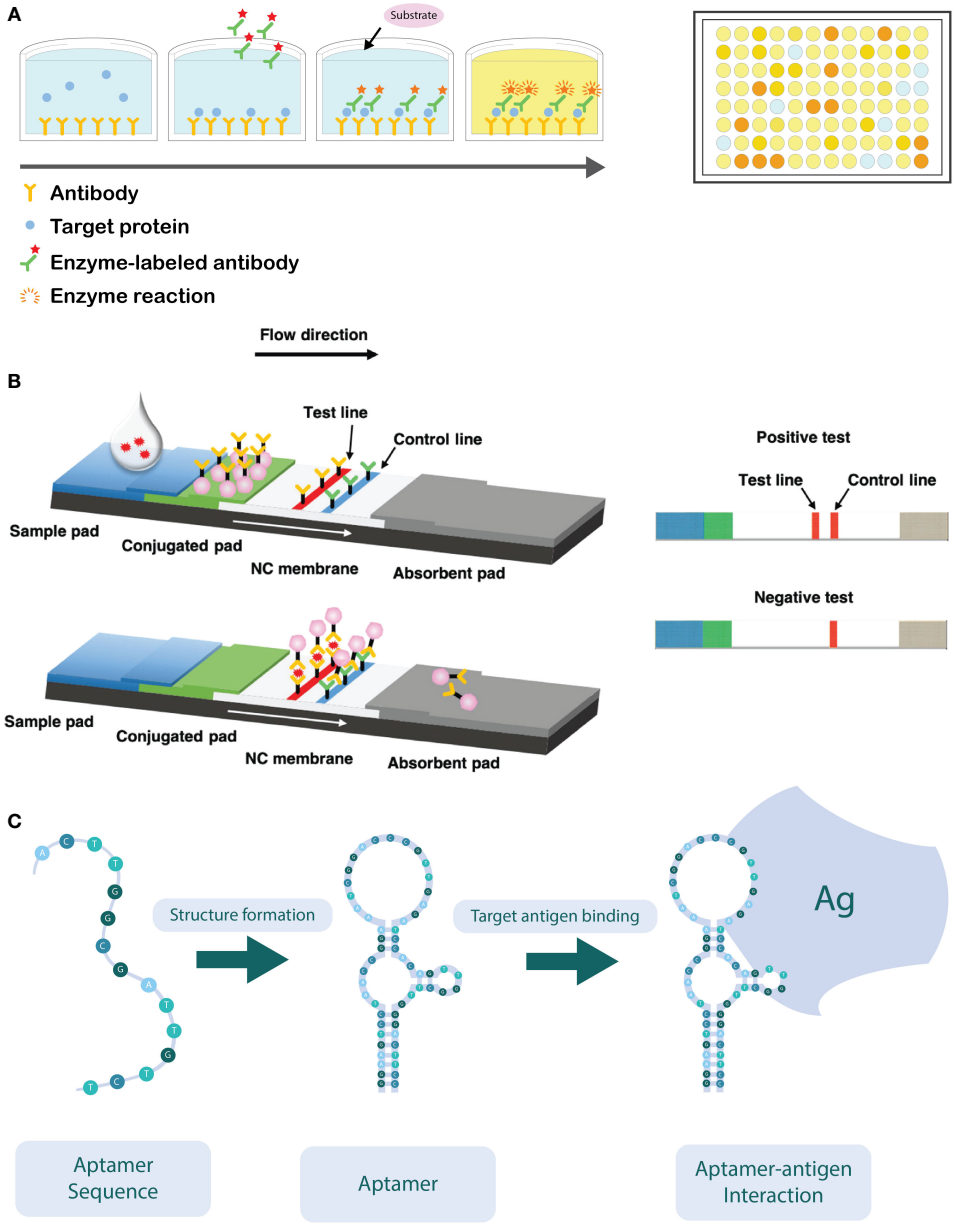
**(A)** Schematic overview of a sandwich ELISA assay. The wells are coated with an antibody which targets the antigen. The antigen of interest in the sample is then added and binds to the capture antibody. Next, the detection antibodies are added, which target a different epitope of the antigen. The detection antibodies are labeled with an enzyme, capable of converting a colorless substrate to generate a colorimetric signal. ELISA reactions are typically performed in a 96-well plate. **(B)** Schematic overview of the working principle of the lateral flow immunoassay (LFIA). After application of the sample on the sample pad, it flows in the direction of the absorbent pad due to capillarity and passes through the conjugate release pad, where the labelled detector antibodies can bind to the target analyte. Next, the sample will continue to flow towards the test and control lines, where the analyte (coupled to the detector antibody) will bind to specific (secondary) antibodies immobilized in the test zone. The excess of unbound detector antibodies will flow towards the control zone, where they are bound to immobilized antibodies specific for the detector antibody. Aggregation of the labelled detector antibodies in both test and control zone can be visually observed as illustrated on the right-hand side of the figure (Adapted from Hsiao et al., 2021). **(C)** Schematic illustration of how the oligonucleotide sequence self-hybridizes into its functional conformation. In its functional conformation, the aptamer is able to bind to its target antigen (Ag) (Adapted from Sun et al., 2014).

By far, the most used immunological technique is the Enzyme Linked Immunosorbent Assay (ELISA) (**Figure 3A**). First developed in the 1970s, it has become a well-established method that is commonly used in detection of microbial pathogens around the world. It is widely used because it is a fast technique and amenable for automation and high-throughput screenings (Alvarez, 2004; Posthuma-Trumpie et al., 2009; Martinelli et al., 2015). Several ELISA formats have been developed, including direct, indirect, sandwich, and competitive ELISA assays (Alhajj and Farhana, 2023), all sharing the same principle, i.e. the use of specific antibodies that are conjugated with an enzyme that can convert a colorless substrate into a colored product. The color formation is proportional to the quantity of the target antigen, and hence, the target pathogen, in the sample (Kumar et al., 2008; Atmar, 2014).

The sensitivity and specificity of ELISA assays can vary considerably between different assays and is largely dependent on the type (e.g., direct, indirect, sandwich, or competitive ELISA), the antibodies used (monoclonal or polyclonal), the conjugated enzyme, and the corresponding substrate. In addition, the matrix can also have a large impact on specificity and sensitivity of ELISA assays, a problem commonly known as “matrix interference” (Wang et al., 2007). This can result in an increased number of false-positives (due to reduced specificity) as well as false-negative results (due to reduced sensitivity).

For instance, indirect ELISA is usually more sensitive because multiple enzyme-labeled secondary antibodies bind with high specificity to the primary antibody bound to the antigen, which results in signal amplification (Katikireddy and O’Sullivan, 2011). Other strategies have been developed to increase sensitivity. Examples include the use of avidin-biotin complexes, where biotinylated antibodies allow for the binding of several avidin complexes linked with an enzyme (Atmar, 2014), or the peroxidase-antiperoxidase approach (PAP), where a PAP complex containing several HRPs is coupled to a secondary antibody (Katikireddy and O’Sullivan, 2011).

ELISA is relatively simple to perform, with the time needed to perform the assay being in the range of one to several hours, if no prior enrichment step is performed (Bari and Kawasaki, 2014). The use of multi-well plates of various formats facilitates the simultaneous testing of several samples and can even be automated (Posthuma-Trumpie et al., 2009). However, some drawbacks are associated with the use of ELISA. Due to poor chemical and physical stability of antibodies, they require refrigeration for storage and special buffers. In addition, the production of novel antibodies can be rather complicated and expensive (Sakamoto et al., 2018).

Since the development of ELISA assays, it has found wide application in agriculture for detection of plant pathogens. For instance, ELISA assays are recommended by EPPO guidelines to test for the presence the of viruses on grafting material of fruit trees (Boonham et al., 2014). And even to date, new ELISA assays are developed to detect plant pathogens. For example, Gorris et al. (2021) recently described the development of a double antibody sandwich ELISA (DAS-ELISA) for detection of the plant pathogen *Xylella fastidiosa*. This DAS-ELISA assay proved to be very specific (no false-positives were observed on real samples) and reliable, with a sensitivity of 10^4^ CFU/ml. Wu et al. (2011) described the development of a monoclonal antibody specific for the detection of Odontoglossum ringspot virus using a triple antibody sandwich ELISA assay. The specificity was evaluated against seven other plant viruses, showing no cross-reactivity.

### 4.2 Lateral flow immunoassays (LFIA)

The lateral flow immunoassay (LFIA) is another commonly used immunological assay for plant pathogen detection (López-Soriano et al., 2017). Such assays consist of nitrocellulose membrane strips that are contained in a plastic receptacle. The analyzed sample is applied on the sample application area (**Figure 3B**). The sample passes through the (primary) antibody conjugate release pad by capillary forces, which allows the antibodies to bind to the target antigen in the sample. Subsequently, the sample flows towards the test area of the device. In the first test zone, antibodies that are specific for (another epitope of) the target antigen are irreversibly bound to the nitrocellulose membrane, while in the second test zone, antibodies against the primary antibody are spotted that serves as a positive control. The primary antibodies present in the release pad are generally labelled with colloidal gold nanoparticles or latex particles, which upon aggregation in the test zone allow for a visual detection of the presence (or absence) of the target antigen (Posthuma-Trumpie et al., 2009; Koczula and Gallotta, 2016; López-Soriano et al., 2017; Singh and Singh, 2020). Although LFIAs are usually developed for detection of a single analyte, in medical diagnostics multiplex LFIAs are developed that are able to detect multiple analytes in a single assay. Such multiplexing can be achieved either by changing the device architecture (e.g., by adding more test zones on the device) or by monitoring signals that are discriminatory for each analyte (e.g., by using differently colored labels) (Anfossi et al., 2019).

LFIA is easy to use, portable, low-cost, and can provide results in around 10 minutes, excluding sample preparation, making them an ideal point-of-care diagnostic method (Boonham et al., 2008; López-Soriano et al., 2017; Singh and Singh, 2020). Such tests usually have a long shelf life, guaranteeing stability for 12-24 months at room temperature (Ahmed et al., 2020). The tests are primarily meant for a qualitative detection of the target pathogen and provide the results by visual observation of a colored line in the test zone. However, semi-quantification is possible if combined with a sensor (Posthuma-Trumpie et al., 2009). A drawback is that only a limited amount of sample can be loaded onto the sample application area, limiting its sensitivity (Koczula and Gallotta, 2016). The method is also limited to liquid samples. For testing solid samples or complex matrices such as soil or plant material, the LFIA requires a sample pretreatment step to extract the relevant target antigens (Posthuma-Trumpie et al., 2009).

The analysis speed and ease-of-use in the field has led to the development of several LFIAs to detect plant pathogens. For instance, a polyclonal LFIA has been developed for detection of *Xanthomonas campestris* pv. *musacearum* (Hodgetts et al., 2015). This assay enabled detection of all *X. campestris* pv. *musacearum* strains, but also showed cross-reactivity to *X. axonopodis* pv. *vasculorum.* The sensitivity of this test was established at 10^5^ CFU/ml. An analogous test to detect *Xanthomonas arboricola* pv*. pruni* showed similar specifications, with high specificity (only showing cross- reactivity against *X*. *arboricola* pv. *corylina*) and with a sensitivity of 10^4^ CFU/ml (López-Soriano et al., 2017). EPPO also recommends the use of LFIAs for detection of plant pathogenic viruses, such as Tomato spotted wilt virus, Impatiens necrotic spot virus, and Watermelon silver mottle virus. However, EPPO also advises that positive LFIA tests need additional confirmation by ELISA- or PCR-based methods, to avoid false-positive results (EPPO, 2004).

### 4.3 The use of aptamers as alternative for antibodies

An alternative for the use of antibodies consists of the use of aptamers. Aptamers are short oligonucleotides (DNA or RNA) of 10-100 nucleotides in size, with a specific three-dimensional conformation and a high affinity to the target analyte/pathogen (**Figure 3C**) (Toh et al., 2015; Zhao et al., 2018). Briefly, such aptamers can be used in a similar way as antibodies targeting a specific antigen. The use of aptamers shows high potential to replace antibodies, due to their ease of production, low-cost, resistance to degradation, small size and ease of labelling. In that regard, aptamers can replace antibodies in the development of enzyme-linked apta-sorbent assay (ELASA) and lateral flow devices (Toh et al., 2015). The use of aptasensors is still inhibited by some factors, e.g., selectivity and affinity are strongly influenced by conditions such as temperature, pH, ionic strength, and viscosity of the sample (Shahdordizadeh et al., 2017). Although aptamer-based selection is not yet commonly used for plant pathogen detection, a few studies demonstrated its value in for instance detection of apple stem spitting virus and soybean rust fungi (Komorowska et al., 2017; Krivitsky et al., 2021).

## 5 Nucleic acid-based assays

Nucleic acid (DNA or RNA) sequences make excellent molecular targets for the detection and identification of (pathogenic) microorganisms, and can be used for viral, fungal and bacterial targets. The presence of a genetic sequence that is unique to the target pathogen, can be detected by polymerase chain reaction (PCR), isothermal amplification techniques, and hybridization-based techniques. Many variant techniques exist, but the most commonly used methods to detect plant pathogens are discussed below in more detail. Alternatively, a sequencing method could be employed to detect unique DNA or RNA sequences, which is discussed in more detail in section 6.

Common for many nucleic acid-based, and especially PCR-based techniques, is that the extracted DNA (or RNA) of the target pathogen in the sample must be of high purity, because most nucleic acid-based assays are sensitive to inhibitors that might be coextracted. Especially in the cases of plant pathogen detection, samples are often composed of difficult matrices, such as plant tissue or soil. The presence of polysaccharides, phenolic compounds, humic acids, or heavy metals in such samples reduces the performance of nucleic acid-based assays (Lievens and Thomma, 2005; López et al., 2009). However, a wide variety of different extraction protocols exist for obtaining pure DNA. These can range from very simple (e.g., commercially available kits) to quite complicated, such as DNA extraction procedures specifically developed for difficult matrices that also include pretreatments (e.g., with liquid nitrogen), additional enzymatic steps, etc. (López et al., 2009). Lastly, the use of some DNA extraction methods is unsuited for point-of-care applications as they are difficult to carry out outside a laboratory. Therefore, DNA extraction methods have been developed that require minimal equipment. Examples of such point-of-care DNA extractions methods are reviewed elsewhere (Lau and Botella, 2017; Paul et al., 2020).

Another common problem associated with nucleic acid-based assays is that they have difficulties with differentiating viable microorganisms from non-viable, as DNA can stably remain in the sample for a considerable time after the organisms have died (Lievens and Thomma, 2005; López et al., 2009; Narayanasamy, 2011). There are ways to circumvent this problem, such as targeting RNA specifically, as it usually degrades rapidly outside the cell. However, robust and efficient RNA extraction from difficult sample matrices such as those taken for plant pathogen detection is not always straightforward, and in some matrices RNA is stable longer than expected (Lievens and Thomma, 2005; Kralik and Ricchi, 2017; Schostag et al., 2020). Alternatively, “live/dead probes” could be used. These are based on compounds that cannot pass the (intact) cell membrane, such as propidium monoazide (PMA) and ethidium monoazide (EMA) and can therefore only bind free DNA. After binding, the free DNA is excluded for further amplification, by which only DNA extracted from intact cells, and hence from viable cells, can serve as a template for PCR. Nevertheless, such probes have showed mixed success in differentiating dead from live cells, and are therefore not widely used in plant pathogen detection (Lievens and Thomma, 2005; Kralik and Ricchi, 2017).

### 5.1 Conventional PCR and variants

Polymerase chain reaction (PCR) is a technique used to amplify specific DNA fragments, making use of oligonucleotide primers, a DNA polymerase enzyme, dNTPs and a thermal cycler. Good primer design allows amplification of a specific DNA fragment that is unique for the targeted pathogen. Detection of a PCR fragment with the expected size is used to confirm the presence of the target pathogen (Zhao et al., 2014; Shen, 2019). In a conventional PCR (cPCR) set-up, this is usually assessed with an end-point detection, such as agarose gel electrophoresis. The run time of such analysis can be time-consuming, which is considered as a major drawback of cPCR. Furthermore, the need for opening tubes for gel electrophoresis increases the risk of contaminating the lab environment, reagents, materials or other samples with the amplified product (Maurer, 2011). PCR is a specific and highly sensitive technique. In theory, according to Poisson statistics, the technique is capable of amplifying as low as 3 copies of the target nucleic acids, but in practice the limit of detection is highly dependent on the sample type, efficiency of the DNA extraction, and the efficiency of the amplification, which on their turn are influenced by the PCR set-up and the primer design (Kralik and Ricchi, 2017). The high sensitivity allows to detect low abundant, slow growing, or non-culturable cells. The specificity of PCR largely depends on proper design of highly selective primers, which relies on the availability of genetic information of the target microorganism (Schaad et al., 2003). Primers need to be carefully designed in such a way that only the genetic sequences of interest are amplified, and false-positive results are avoided. As such sensitivity and specificity of a PCR assay should always be evaluated and validated case by case (López et al., 2009). In addition to a high sensitivity and specificity, PCR is also considerably faster than the conventional culture-based methods; results can be obtained in a matter of hours (Mandal et al., 2011; Priyanka et al., 2016; Ferone et al., 2020).

However, PCR-based methods also have some drawbacks: (i) it is sensitive to PCR inhibitors that may be present in the sample, which results in false-negative results; (ii) it cannot distinguish viable from non-viable cells; (iii) it requires a laboratory environment; (iv) PCR is unable to amplify RNA targets, e.g., for detection of viral pathogens; (v) quantification of the target pathogen is not possible in conventional PCR; and (vi) the high sensitivity can lead to an increased risk of obtaining false-positive results, due to non-specific amplification or contamination due to carry-over from other samples during handling (Ward et al., 2004; López et al., 2009). Furthermore, in case multiple pathogens should be detected in a single sample, separate PCR procedures should be used, which can quickly become expensive to use (Liu et al., 2019). To overcome some of these disadvantages, several PCR variants have been developed that increase its application potential for the detection of plant pathogens. The most commonly used variants are discussed below.

A first PCR-variant, i.e. reverse transcriptase PCR (RT-PCR), can be used on RNA targets, which is useful for the detection of viable cells and RNA viruses (López et al., 2009). RT-PCR includes a reverse transcription step, in which the RNA template is copied into a complementary DNA (cDNA) strand. The cDNA is subsequently used as a template in a conventional PCR reaction, usually followed by gel electrophoresis to detect the amplified product (Li and Hartung, 2007). In this way expressed genes (produced by viable target microorganisms) or viral RNA can be detected.

Multiplex PCR utilizes two or more primers sets that are designed to target different genetic sequences within the same PCR reaction. This allows simultaneous detection of multiple target pathogens. However, the primers must be carefully designed to avoid interference between primers of different primer sets and should also amplify DNA targets with different sizes that allow discrimination of the expected PCR-products by gel electrophoresis (Shen, 2019). Consequently, the number of target pathogens that can be amplified simultaneously is rather limited. Although the initial development of a multiplex PCR reaction can take considerable effort, once validated it increases the diagnostic capabilities of the method as it saves time, effort, and costly reagents for each PCR run (Elnifro et al., 2000). One of the downsides of multiplex PCR, however, is that they are more prone to non-specific DNA amplification, and consequently false-positive results, due to the presence of multiple primer pairs (Lau and Botella, 2017). Furthermore, multiplexing may compromise the sensitivity, as usually a certain target is amplified more efficiently and can outcompete amplification of other targets (Elnifro et al., 2000; Okubara et al., 2005). For instance, it has been shown that a multiplex PCR assay to detect two *Phytophthora* spp. has a detection limit of 10-100 pg DNA/µl, while the individual singleplex PCRs had a detection limit of 1 pg DNA/µl (Ippolito et al., 2004). A good example that illustrates the advantages of using multiplex PCR (mPCR) to detect multiple plant pathogens is the study of Cui et al. (2016), in which an mPCR is developed to detect 6 major bacterial pathogens of rice. High specificity was demonstrated by checking for false-positive or false-negative results with ~120 closely related non-target strains and ~30 target strains, respectively. Considering that 6 pathogens can be detected in one test, this mPCR is considered as a time- and cost-saving method. However, it also illustrates some limitations, as the sensitivity to detect all pathogens in rice seed samples was established at 10^5^ CFU/ml for the multiplex assay, whilst in the individual PCR reactions the detection limit was at 10^3^ CFU/ml. Another example of a mPCR assay was developed by Chavhan et al. (2023) for detection of commonly occurring cotton (*Gossypium* spp.) pathogens, including fungal, bacterial and viral targets. In other assays, a reverse transcription step is included, allowing detection of RNA viruses as well. For instance, Adkar-Purushothama et al. (2010) developed such a test for the simultaneous detection of Citrus tristeza virus (CTV) and *Candidatus* Liberibacter species from citrus plants.

Another variant is nested PCR (nPCR), which relies on two successive amplification rounds. The first round uses a set of (outer) primers that amplify a larger region of the target DNA. The PCR-product of the first round is subsequently used as a template in the second amplification round, using (inner) primers that anneal to a sequence internal to the sequence amplified by the first primer set (Shen, 2019). The technique usually results in a more sensitive detection, due to the high (combined) total number of PCR-cycles. The high sensitivity of nPCR makes it especially useful for detection of low-titer pathogens, as exemplified in the nPCR assay developed for detection of Phytoplasmas, *Phytophthora* and citrus tristeza virus in a wide range of different plant samples (Adkar-Purushothama et al., 2011; Engelbrecht et al., 2013; Nair and Manimekalai, 2021). In addition, nPCR generally has a higher specificity compared to traditional PCR because it is very unlikely that non-specific PCR products from the first amplification round also contain binding sites for the inner primers (López et al., 2009; Shen, 2019). On the other hand, the technique is more prone to carry-over contamination between the successive PCR reactions. Furthermore, nPCR is more costly and labor-intensive, due to the fact that essentially two PCR reactions need to be carried out per test (López et al., 2009; Mancini et al., 2016; Nair and Manimekalai, 2021). These disadvantages can be reduced by utilizing multicompartment reaction vessels, as this eliminates the need for post-amplification manipulations and reduces the risk of carry-over contamination (López et al., 2009).

### 5.2 Quantitative PCR

Quantitative PCR (qPCR), also referred to as real-time PCR, operates on the same working principle as conventional PCR with the main difference being that the amplified DNA is measured during the PCR-reaction in real time instead of an end-point detection as described above (Shen, 2019). Adding fluorescent dsDNA-binding dyes or sequence-specific probes allows to assess the amount of amplified DNA after each cycle, since the fluorescence intensity is a measure for DNA amplification (Postollec et al., 2011). The use of dsDNA-binding dyes, such as the frequently used SYBR green dye, is cheaper, but has the drawback that such dyes also bind with non-specific amplification products. To check for non-specific amplification, a melting curve (T_m_) analysis can be carried out after the end of the qPCR (Okubara et al., 2005; Mirmajlessi et al., 2015). Although not straightforward, in some cases the T_m_ analysis even allows for multiplexing if the generated amplicons differ in size/nucleotide composition. For example, a multiplex real-time RT-PCR was developed based on melting curve analysis to detect 4 different variants of Grapevine leafroll-associated virus 3 (GLRaV-3) in vineyards (Bester et al., 2012). In addition, different types of sequence-specific probes can be used in qPCR, including TaqMan probes, molecular beacons and scorpion probes. Such probes show higher specificity compared to dsDNA-binding dyes, since fluorescence is only emitted when the probe hybridizes with its target sequence. When different fluorescently labeled probes targeting different targets are used, it also allows (limited) multiplexing, with a maximum of 4-6 genetic targets per reaction (Rajagopal et al., 2019). Similar difficulties as in conventional multiplex PCR apply in regard to sensitivity and specificity (Okubara et al., 2005). The drawback of using probes is that they are generally more expensive than their SYBR green counterpart (Okubara et al., 2005; Mirmajlessi et al., 2015). Interestingly, it has been shown that the PCR cycle in which the fluorescent signal exceeds a certain threshold (called threshold cycle C_T_ or quantification cycle C_Q_) is inversely proportional to the logarithm of the target DNA that was originally in the sample. In this way, the DNA concentration, and hence, the target pathogen present in a sample, can be quantified by making use of a standard curve in which C_T_ values are determined for samples with known concentrations of DNA template (Shen, 2019).

The use of qPCR for plant pathogen detection has several advantages. The technique is more sensitive than conventional PCR, which can be mainly ascribed to two reasons: (i) instrumental fluorescence measurements are more sensitive than visualization of a DNA-fragment after gel electrophoresis; and (ii) qPCR targets are typically short (70-150bp), which are amplified more efficiently (Smith and Osborn, 2009; Schena et al., 2013; Mirmajlessi et al., 2015). The increased sensitivity makes it a valuable tool for early detection of pathogens, even before disease symptoms are visible (Okubara et al., 2005). In addition, the fact that gel electrophoresis is not required, leads to a faster analysis time, and makes it more prone to automation (Postollec et al., 2011; Law et al., 2015). Furthermore, pathogenic RNA viruses can also be detected and quantified by adding a reverse transcription step (Boonham et al., 2004; Bester et al., 2012). However, the largest asset is the possibility of quantification of the pathogen. This enables to determine action thresholds in the field, i.e. the pathogen level at which treatment is required, leading to less frequent application of chemical pesticides, and ultimately to a more efficient and sustainable disease management strategy (Okubara et al., 2005). Although quantification is possible, the assays are more frequently used for qualitative purposes, i.e. presence or absence of specific microorganisms.

Several examples of qPCR methods for plant pathogen detection described in literature point to the advantages over cPCR (Narayanasamy, 2011). For instance, it was shown that a qPCR method to detect *Phytophthora cryptogea* was able to detect 50 zoospores, while a cPCR with the same primers could only detect 5000 zoospores (Minerdi et al., 2008). A qPCR developed for detection of *P. cactorum* in strawberry samples also showed a high specificity and sensitivity and was able to detect up to 10 zoospores/g plant material (Verdecchia et al., 2021). Also for bacterial plant pathogens, several qPCR methods have been developed. For instance, detection of rhizogenic *Agrobacterium* strains in irrigation water of tomato greenhouses showed a sensitivity of 1 CFU/ml water (Bosmans et al., 2016).

### 5.3 Digital droplet PCR

**Figure 4:**
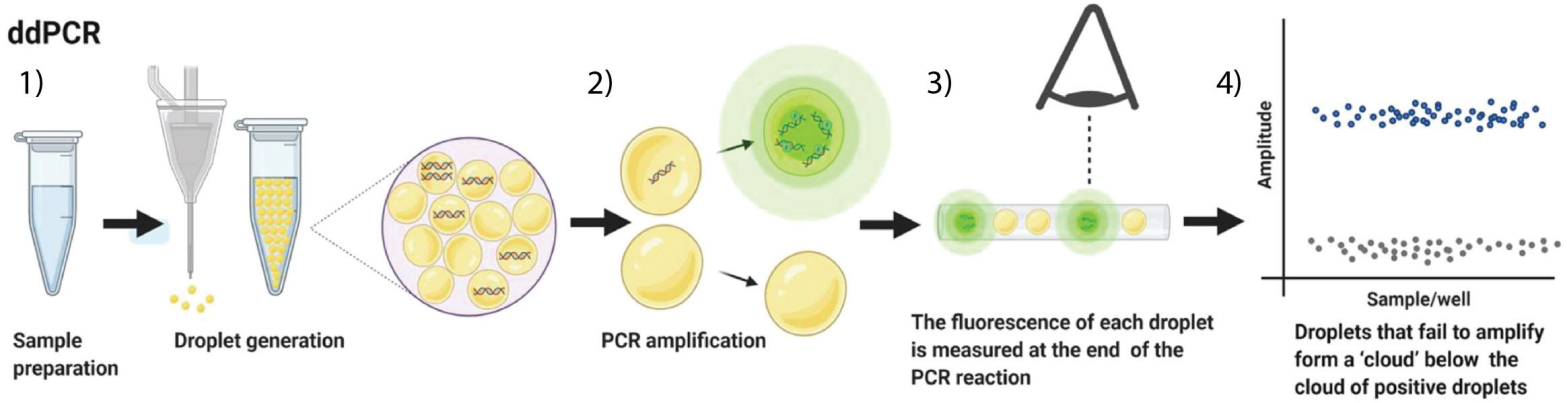
Schematic representation of a digital droplet PCR workflow. From left to right: The DNA sample is prepared by generating water-in-oil droplets containing template and the necessary PCR reagents and dyes (1); The droplets are thermally cycled until the PCR reactions reach their end-point (2); The presence of an amplicon (and hence target DNA in the sample) in each droplet is visualized by dsDNA binding dye or by sequence-specific probes and is detected in a microfluidics device (3). Fluorescent signals are processed to detect and quantify the number of pathogens in the sample (4) (Adapted from Kokkoris et al., 2021).

Digital droplet PCR (ddPCR) relies on the same principles as conventional PCR, however, it allows for the absolute quantification of nucleic acids in a sample (Hindson et al., 2011). In this technique, the DNA in a sample is partitioned in about 20.000 miniscule water-in-oil-droplets, with ideally each droplet containing either no or a single copy of template DNA (Hindson et al., 2011; Hayden et al., 2013; Chen et al., 2021). The droplets will each act as an individual PCR reaction vessel, in which a DNA region that is specific for the target pathogen (if present) is amplified. Addition of fluorescent probes or intercalating dyes enables to detect whether a PCR reaction has occurred. The resulting droplets are subsequently passed one by one through a microfluidics system to determine the fluorescence (**Figure 4**) (Hindson et al., 2011; Zhao et al., 2016). With the use of Poisson statistics, the number of droplets that contain an amplicon can be used to determine the amount of template DNA present in the original sample (Hindson et al., 2011; Hoshino and Inagaki, 2012; Chen et al., 2021).

Digital droplet PCR has some advantages over real-time PCR. First and foremost, there is no calibration curve needed for quantification, and the DNA present is quantified in a direct way. This makes the quantification more reliable, as real samples can have different amplification efficiencies than those obtained in setting the calibration curve (Hayden et al., 2013; Taylor et al., 2017). In addition, ddPCR is more sensitive and more resistant to PCR-inhibitors compared to qPCR. As ddPCR is based on end-point detection, this technique is less reliant on the amplification efficiency of the PCR reaction itself, as is the case with qPCR (Taylor et al., 2017). A study evaluating the sensitivity and resistance to PCR-inhibitors in qPCR and ddPCR methods to detect *Xanthomonas citri* sp. *citri* demonstrated that a limit of detection for qPCR and ddPCR was obtained of 36 CFUs/20 μL and 5 CFUs/20 μL, respectively. It was also shown that ddPCR performed better than qPCR in the presence of increasing concentrations of citrus leaf extract to evaluate the sensitivity to inhibitors (Zhao et al., 2016). Altogether, this makes ddPCR more useful for the analysis of complex sample matrices, such as soil, which is an added value in the field of plant pathogen detection (Hoshino and Inagaki, 2012; Chen et al., 2021). On the other hand, currently ddPCR is still more expensive than qPCR. The cost per test is approximately 2.3-fold higher (Hindson et al., 2011; Morcia et al., 2020; Maheshwari et al., 2021). The assay also takes approximately 2-3 times longer compared to qPCR, due to a more complicated workflow, the fact that the reactions need to reach their end-point and the microfluidics fluorescence measurements (Hindson et al., 2011; Morcia et al., 2020; Maheshwari et al., 2021). Lastly, the ddPCR has a lower dynamic range for quantification as compared to qPCR, as the limited number of droplets can hinder accurate quantification once they approach saturation with target DNA (Ricchi et al., 2017; Morcia et al., 2020).

Recent examples of ddPCR methods developed for plant pathogen detection include a ddPCR assay for detection of *Xylella fastidiosa* (Dupas et al., 2019), *Acidovorax citrulli* (Lu et al., 2020), *Tilletia controversa* (Liu et al., 2020), and an RT-ddPCR for detection of peach latent mosaic viroids extracted from infected peach leaves (Lee et al., 2021). ddPCR is also suitable for multiplexing applications although there are only few examples described in literature focusing on plant pathogen detection, including a multiplex ddPCR assay for detection of *Candidatus Liberibacter asiaticus* and *Spiroplasma citri* (Maheshwari et al., 2021). Altogether these studies showed that ddPCR is a sensitive and robust technique that is highly valuable for monitoring low titer pathogens in complex samples.

### 5.4 Isothermal nucleic acid amplification

Although PCR-based methods for plant pathogen detection are common practice, their use for in-field diagnostics is generally limited by the requirement of a thermal cycler, as well as highly purified DNA (Lau and Botella, 2017). The use of isothermal amplification techniques proposes itself as a valuable alternative. Isothermal amplification techniques utilize amplification mechanisms that do not require thermal cycling equipment, but instead rely on the use of strand-displacing DNA polymerases. Simple equipment, such as heating blocks, can be used instead of thermal cyclers to perform the assays (Ivanov et al., 2021). Another advantage of isothermal amplification is that these techniques do not require highly purified DNA. Several detection techniques use isothermal amplification, of which the loop-mediated isothermal amplification (LAMP) and recombinase polymerase amplification (RPA) will be discussed in more detail below (Becherer et al., 2020). Although a variety of other isothermal amplification techniques exist, we have chosen to only discuss LAMP, as it is the most commonly used isothermal amplification method (Gomez-Gutierrez and Goodwin, 2022), and RPA, because its use has seen a rapid increase the last decade (Li et al., 2018). For a more exhaustive summary on other isothermal amplification techniques, we refer the reader to the review of Oliveira et al. (2021).

#### 5.4.1 Loop-mediated isothermal amplification

**Figure 5:**
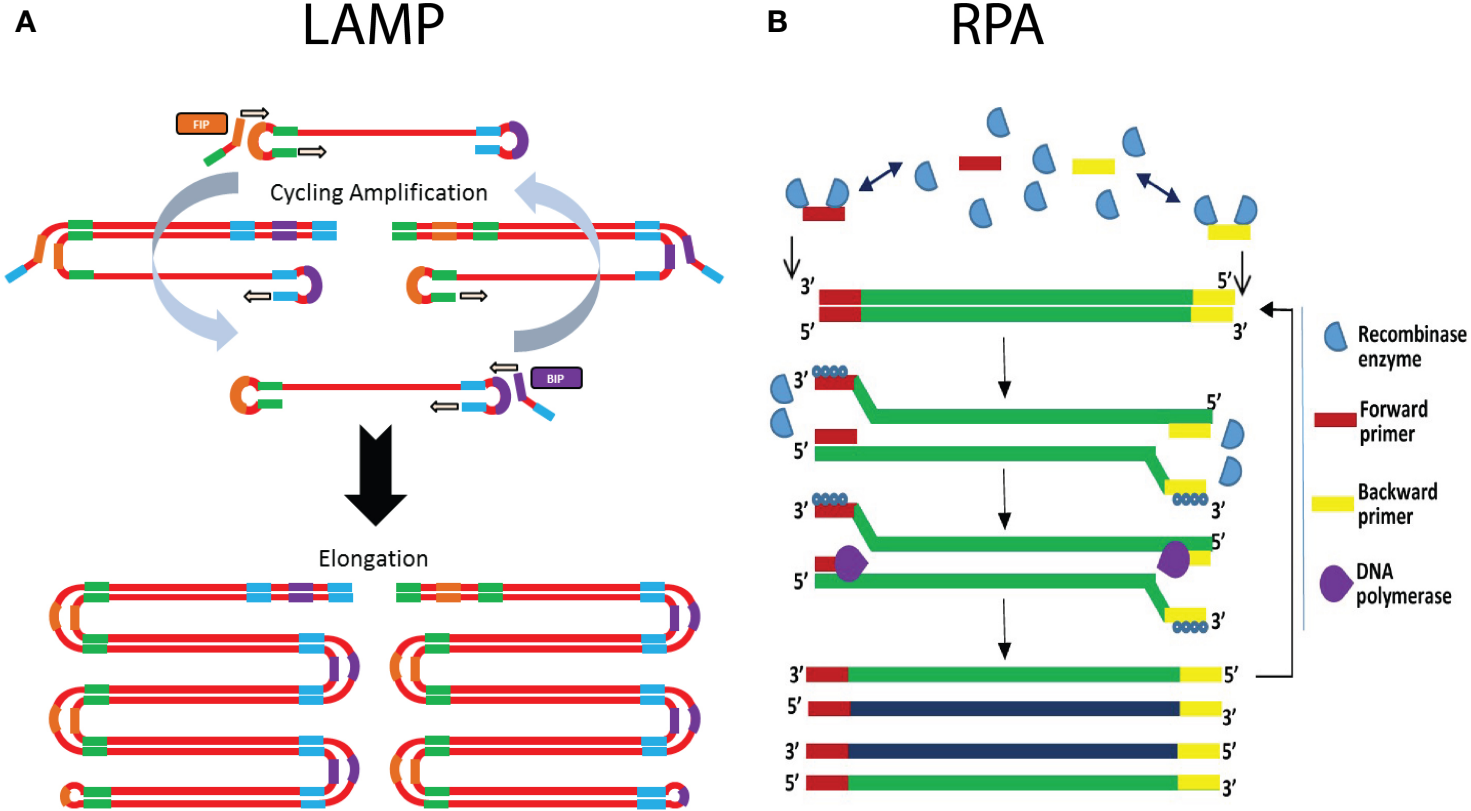
Schematic illustration of the working principle of isothermal amplification methods LAMP and RPA. **(A)** In LAMP, the use of self-complementary forward and reverse primers results in the formation of a characteristic dumbbell-like structure. This serves as a template for isothermal amplification that is initiated by annealing of a mixture of self-complementary primers. This results in the generation of an increasing number of additional priming sites, ultimately leading to concatamers of the dumbbell DNA structure. **(B)** On the right hand side the RPA mechanism is shown, with the recombinase enzymes that bind the forward and reverse primers, which subsequently scans the template DNA for complementary sites. Upon finding the complementary site, the primer binds to its complementary sequence though strand invasion. The polymerase generates a new complementary DNA strand starting from the primers, thereby displacing the original DNA strand. The use of a strand-displacing DNA polymerase avoids the need for denaturation. (Adapted from Lmstanfield (2014), Lmstanfield at English Wikipedia, CC BY-SA 3.0 , via Wikimedia Commons and Obande and Singh, 2020).

Loop-mediated isothermal amplification (LAMP) ranks as the most cited isothermal amplification assay in literature (Becherer et al., 2020). Briefly, this technique relies on the utilization of at least four primers, two inner and two outer primers, in combination with a strand-displacing DNA polymerase (**Figure 5A**). The combination of primers and strand-displacing polymerase activity leads to the formation of a piece of single stranded DNA, which at both ends forms a dumbbell-like structure, due to the intramolecular complementarity of the inner primers. These dumbbell structures are target for binding of the inner primers and outer primers. Due to this self-priming ability in combination with the strand-displacing polymerase, new amplified DNA is generated continuously. The resulting reaction products consist of long concatemers of the target DNA region (Craw and Balachandran, 2012; Lau and Botella, 2017; Becherer et al., 2020; Ivanov et al., 2021). The reaction is carried out at a constant temperature of 60-65°C and usually generates results within 30 minutes (sample preparation excluded), depending on the target as well as the primers that are used (Becherer et al., 2020; de Paz et al., 2020; Ivanov et al., 2021). For instance, the analysis time can be reduced by adding two additional primers, which target the loops of the dumbbell structure, providing for two additional polymerase initiation sites. Multiple methods can be used for the detection of LAMP-based amplification products, including gel-electrophoresis, measurement of turbidity due to precipitation, fluorescent DNA binding dyes, sequence-specific fluorescent probes, and lateral flow devices (Mori et al., 2001; Craw and Balachandran, 2012; Naidoo et al., 2017; Panno et al., 2020). For more details on the ins and outs of LAMP, we refer to other review papers (Notomi et al., 2000; Becherer et al., 2020).

The advantages of using LAMP primarily consist of the fact that simple equipment can be used for isothermal amplification and measurement, which enables in-field application. Depending on the primers and the DNA extraction procedure that is used, analysis time is very short (<30 minutes). LAMP assays are more robust in the presence of PCR inhibitors, and for this reason many LAMP protocols only require crude extracts of the samples. Minimal processing procedures for LAMP include grinding of plant tissue, addition of lysis buffer, boiling the samples, etc. The fact that minimal sample preparation is required, leads to a reduced analysis time and cost. On the other hand, when sample preparation also includes DNA extraction, the sensitivity of the assay is increased. A comparative study which evaluated LAMP with PCR-based assays for detection of *Alternaria solani* indicated that the sensitivity of the LAMP-assay falls between cPCR and qPCR (Khan et al., 2018). One of the main disadvantages is that in general, LAMP is only useful for qualitative assays. However, recent studies suggest that quantification could be possible using a strategy similar to qPCR, in which the time to reach a signal threshold is correlated with the initial amount of DNA/pathogen present in the sample (Nguyen et al., 2020). This doesn’t limit the in-field application, as it has been shown that the signal can be quantified using a smartphone camera, or a portable fluorescence measurement and heating device such as the Optigene Genie II (Becherer et al., 2020; Enicks et al., 2020; Nguyen et al., 2020). Nevertheless, it is generally recognized that the quantification accuracy of LAMP is inferior to that of qPCR, especially in the lower end of the quantification ranges (Moehling et al., 2021). Another drawback of LAMP is the complex primer design process, although primer design tools exist for this purpose, e.g., “LAMP Designer” or “NEB LAMP” (Jia et al., 2019). Non-optimal primers can lead to formation of non-specific products and primer dimers. Since there is no way to discriminate the fluorescent signal resulting from specific or non-specific amplicons, this could lead to false-positive results (Ivanov et al., 2021). In these cases it might be advisable to make use of sequence-specific detection methods such as fluorescent probes, although this would require the utilization of a more expensive fluorescence measurement device. Another risk is the possibility of carry-over contamination with the amplified product during sample handling and post-amplification visualization, as discussed above (Gomez-Gutierrez and Goodwin, 2022). Therefore, closed tube reaction visualization is often preferred. In theory, multiplex LAMP assays could be possible, but are generally too difficult to develop due to the complexity of the primer design and the risk of non-specific amplification (Craw and Balachandran, 2012; Becherer et al., 2020; Ivanov et al., 2021).

LAMP assays have been developed for detection of a variety of plant pathogens, including bacterial, fungal and viral pathogens; the latter in combination with a reverse transcription step if needed (Craw and Balachandran, 2012; Ghosh et al., 2015; Mancini et al., 2016; Li et al., 2019; Panno et al., 2020; Ivanov et al., 2021). Just to mention a few examples, LAMP was successfully used for the detection of *Dickeya dianthicola* on potato plants, using a SYBR Green-based LAMP assay. The detection limit of this assay on artificially spiked plant extract was established at 1 pg/µl (Ocenar et al., 2019). This method requires minimal sample preparation, consisting of the use of a lysis buffer with ball bearings for a fast DNA extraction. Another example involves an RT-LAMP assay for detection of the peach latent mosaic viroid, with a sensitivity that is 100-fold higher as compared to the conventional RT-PCR protocol (Boubourakas et al., 2009). Lastly, Paul et al. (2021) developed a multiplex assay for the detection of *Phytophthora infestans* and tomato spotted wilt virus using a smartphone-based detection system that integrates LAMP. Rapid DNA and RNA extraction was performed with microneedle patches, and the assay takes less than 30 minutes from nucleic acid extraction to results.

#### 5.4.2 Recombinase polymerase amplification

Recombinase polymerase amplification (RPA) is a technique which involves the use of a DNA-recombinase, primers, nucleotides, single stranded DNA (ssDNA)-binding proteins, and a strand-displacement polymerase enzyme (Piepenburg et al., 2006; Lobato and O’Sullivan, 2018). In short, the recombinase proteins bind to the primers, and the resulting complex will scan the DNA template for the complementary (target) sequence (**Figure 5B**). Once a match is found, the recombinase-primer complex will bind to the complementary sequence through strand invasion, followed by displacement of the complementary strand. The ssDNA binding proteins will stabilize the complex, to prevent the displaced strand to rehybridize with the original template DNA. The strand displacement polymerase elongates the hybridized primer in the presence of dNTPs. The same process occurs in the reverse direction, resulting in the exponential amplification of the target DNA (**Figure 5B**) (Li et al., 2018; Lobato and O’Sullivan, 2018). Strand invasion and elongation is done isothermally, usually in a range of 37-42°C (Lobato and O’Sullivan, 2018; Oliveira et al., 2021). Due to the fact that RPA does not rely on thermal cycling, and that the process happens continuously, it allows for rapid amplification of the target DNA. The reaction usually reaches its end point in 20 minutes, making it one of the fastest nucleic acid amplification techniques (Ivanov et al., 2021). Detection usually occurs at the end-point, either through gel-electrophoresis or by a lateral flow device. The lateral flow device format is especially useful for point-of-care applications, as it allows for rapid detection of the amplification products, by labeling the forward and reverse primer with FAM and biotin labels, respectively. The test strip conjugate release pad contains nanoparticles with conjugated anti-FAM antibodies, whilst the capture line consists of immobilized streptavidin (Lobato and O’Sullivan, 2018; Ivanov et al., 2021). Aggregation of the amplification product at the test line can be visually assessed. There are also real-time variants, which use fluorescent probes to measure the amplification in real time (Li et al., 2018; Lobato and O’Sullivan, 2018)

Although RPA currently has a small market share, it has seen rapid growth in recent years (Li et al., 2018). This is primarily attributed to the fact that it is comparatively easy to use, has fast analysis times and a low operating temperature (Babu et al., 2018; Lobato and O’Sullivan, 2018; Oliveira et al., 2021). The comparatively lower operating temperature requires a heating block which consumes less power, with some reports of successful amplification occurring using body heat (Li et al., 2018). Because RPA is reported to be more resistant to PCR inhibitors, the technique requires limited sample preparation, considerably reducing sample preparation time. For example, some studies report RPA being successfully applied to crude extracts of plants (Li et al., 2018; Lobato and O’Sullivan, 2018; Ivanov et al., 2021). Altogether, this makes RPA an attractive technique to be used in point-of-care applications. In addition, RPA is highly sensitive, being able to detect 1-10 copies of template DNA in a reaction (Lobato and O’Sullivan, 2018). It can also be combined with a reverse transcription step to target RNA templates, for instance to detect RNA viruses (Babu et al., 2018). Lastly, RPA can be multiplexed by using different primer pairs, or using sequence-specific probes, but just as in regular PCR this process requires careful design (Lobato and O’Sullivan, 2018).

The downside of RPA is that there are reports of primer mismatching in similar DNA sequences, which can cause false-positive results (Babu et al., 2018; Li et al., 2018; Lobato and O’Sullivan, 2018). Therefore, to increase specificity, special care has to be taken when designing the primer. The manufacturer recommends primers of about 30-35 nucleotides long, but there are reports of PCR primers, of around 20 nucleotides in size, obtaining sensitive and specific results (Lobato and O’Sullivan, 2018). In addition, the target area is generally limited to below 500 bp, although amplification products of around 1500-2000 bp have been successfully amplified (Li et al., 2018). Nevertheless, in most cases the target size is restricted to 100-200 bp, which also allows for fast and efficient amplification (Babu et al., 2018).

Several RPA assays have been developed for the detection of plant pathogens, in many cases in combination with lateral flow devices to detect the amplicons (Ivanov et al., 2021). Examples include the detection of *Xylella fastidiosa* in blueberry, where RPA showed similar sensitivity to conventional PCR with detection limits of 1 pg/ul of extracted DNA (Waliullah et al., 2019). An RT-RPA assay was developed for the detection of little cherry virus 2, which showed detection on 100-fold dilutions of crude extract from infected plant samples (Mekuria et al., 2014). Altogether, these studies show that RPA is a rapid and effective detection technique, with a sufficiently high sensitivity that allows for in-field plant pathogen detection.

### 5.5 Hybridization arrays

The ability of DNA strands to hybridize to their respective complementary DNA strands provides for a useful means of detection. The use of labeled sequence-specific probes that hybridize to a specific genetic sequence of a particular pathogen, enables detection of that pathogen. Several hybridization-based assays, such as FISH, southern and northern blotting, etc. exist, but fall out of the scope of this review since they are less suited for detection of plant pathogens. In this review we will focus mainly on hybridization arrays. While most of the techniques discussed so far show limited multiplexing capabilities, hybridization arrays allow simultaneous detection of many (virtually limitless) pathogens (Thies, 2015). Array-based methods (e.g., micro- & macro-arrays, or the Luminex system) involve the immobilization of many sequence-specific capture probes on a solid support (Narayanasamy, 2011). In these assays, a reverse hybridization approach is applied in which the target DNA is labelled instead of the immobilized detector probes, either by fluorescence, radioisotopes or enzymatically. Briefly, DNA in the sample is extracted and universal genes with discriminatory power are amplified and labeled. Next the amplified products are denatured and allowed to hybridize to the detector oligonucleotides on the solid support. Visualization of which detector oligonucleotides that are bound with the labeled amplicon allows to determine which DNA sequences (and hence which target pathogens) are present in the sample.

Two well-known hybridization arrays are micro- and macro-arrays. Although their functioning is largely the same, the main difference between the two lies in the density and amount of immobilized probes, as well as the means of hybridization detection (Narayanasamy, 2011; Aslam et al., 2022). Microarrays are densely packed arrays with thousands of spots of probe DNA less than 200 µm in diameter immobilized on a glass slide (Narayanasamy, 2011). The target DNA is usually labelled with a fluorescent probe and detection is performed by a laser induced fluorescence and a scanning confocal microscope (Bumgarner, 2013). In macro-arrays spots are less densely packed, with spot sizes above 300 µm in diameter immobilized on a nylon membrane (Narayanasamy, 2011). Detection is usually performed with chemiluminescent labels (Lievens et al., 2003; Úrbez-Torres et al., 2015). Lastly, the Luminex xMAP system makes use of detector oligonucleotides that are bound to microbeads. Microbeads covered with a specific detector oligonucleotide can be distinguished from other beads by unique spectral properties (Dunbar, 2006). Contrary to micro- and macro-arrays, which utilize a flat support with localized spots, the Luminex system is a suspension-based array which simplifies the ease of use, has a lower analysis cost, and shows faster hybridization kinetics. Up to 100 different microbeads can be used at the same time, and upon hybridization of labelled target DNA with its respective microbead, the beads are interrogated individually as they pass a set of lasers. While spectral properties allows identification of the specific detector oligonucleotide present on that particular bead, the fluorescent signal indicates whether a complementary sequence, and hence a target pathogen, is present in the sample.

Even though hybridization arrays have the obvious benefit of targeting many pathogens simultaneously, such arrays can only be designed if *a priori* knowledge of the genetic sequences of the target pathogens is available (Bumgarner, 2013). The number of false-positives and false-negative results is highly dependent on the stringency of the hybridization conditions, which requires careful optimization (Sassolas et al., 2008). Furthermore, while microarrays are usually automated, macro-arrays tend to be quite labor- and time-intensive, resulting in an increased cost.

Several arrays have been developed to detect bacterial, fungal and viral plant pathogens (Lievens et al., 2003; Zhang et al., 2008; Narayanasamy, 2011; Charlermroj et al., 2013; Úrbez-Torres et al., 2015; Krawczyk et al., 2017; Bhat and Rao, 2020; Aslam et al., 2022). Commercial kits are also available, such as a macro-array that is able to detect 8 potato viruses simultaneously (BIOREBA). Although several studies demonstrated the value of hybridization arrays for plant pathogen detection in the past, their use in recent years is somewhat declining, in particular due to the decreasing cost of sequencing (Bumgarner, 2013).

### 5.6 CRISPR-Cas-based detection systems

It is generally acknowledged that CRISPR-Cas-based molecular tools have revolutionized molecular biology, and in particular facilitated site-directed mutagenesis in a wide range of different organisms (Doudna and Charpentier, 2014). In addition to its wide use for genome editing, the last few years the potential of CRISPR-Cas systems in molecular diagnostics has been increasingly investigated because of its high specificity and the flexibility that is inherent to this system (Kaminski et al., 2021; Huang et al., 2022). Several strategies have been devised to develop pathogen detection tools that are based on the use of different Cas-variants. In general, these strategies usually rely on DNA extraction and subsequent binding of the Cas protein to a pathogen-specific DNA motif. Such a binding event, and hence presence of pathogenic DNA, results in a measurable signal, which can either be a fluorescent or electrochemical signal, a colorimetric reaction that is visually assessed, or a visual signal on a lateral flow device (Wang et al., 2020). The exact methodology of the wide range of CRISPR-Cas-based detection strategies is beyond the scope of this review, but this is excellently reviewed elsewhere (Wang et al., 2020; Kaminski et al., 2021; Huang et al., 2022).

Several advantages have been attributed to CRISPR-Cas-based pathogen detection systems. In general, they show high potential to be used for point-of-care diagnostics, as they are low cost, highly sensitive and specific, and in principle do not require high-tech equipment (Kaminski et al., 2021; Huang et al., 2022; Karmakar et al., 2022). In addition, the systems are usually able to provide results in a timely fashion, with almost all assays capable of being performed in less than 2 hours (Wang et al., 2020). In general, most CRISPR-Cas-based detection systems have a sensitivity in the picomolar range (Kaminski et al., 2021). However, when combined with preamplification of the target sequences (e.g., by PCR, LAMP, RPA,…), the sensitivity can be significantly increased. In line with the potential of being point-of-care tools, they are often combined with isothermal amplification procedures, such as LAMP or RPA, although PCR-based amplification is also possible (Kaminski et al., 2021). Finally, a major advantage of CRISPR-Cas-based diagnostics is their single-nucleotide specificity, which opens up the opportunity to detect SNPs or strain variants.

Despite the recent progress in CRISPR-Cas-based detection tools, there are still a number of disadvantages and challenges that hamper its wide use in practice. First of all, although there are some strategies to assess more than one pathogen in a single run, the capacity for multiplexing is still rather limited (Gootenberg et al., 2018; Kaminski et al., 2021). Secondly, CRISPR-Cas-based diagnostics usually require tedious sample preparation steps (Benzigar et al., 2021; Huang et al., 2022). Furthermore, in many cases a preamplification step is required to increase the sensitivity and allow detection of low titer pathogens, which results in a significant increase in cost and analysis time (Kaminski et al., 2021). Finally, the downside of the single-nucleotide specificity mentioned above is that a single mutation in the target gene could result in false-negative results, which can be problematic, in particular for detection of viruses with a high mutation rate (Benzigar et al., 2021).

Although until now CRISPR-Cas-based assays are predominantly developed for use in medical diagnostics, recent studies demonstrated the proof-of-concept of CRISPR-Cas-based assays in detection of plant pathogens as well (Sharma et al., 2021; Karmakar et al., 2022). For instance, Aman et al. (2020) developed a CRISPR-Cas-based assay to detect economically important RNA viruses (i.e. Potato virus X and Y, and Tobacco mosaic virus). This assay included a preamplification step using RT-RPA, resulting in the ability to detect picomolar concentrations of viral RNA in the sample within 20 min (excluding RNA extraction). In another study a CRISPR-Cas-based system was developed to detect 5 important apple viruses simultaneously (Jiao et al., 2021). This method also uses an RT-RPA preamplification step and allowed viral RNA detection in the femtomolar range. Analysis of field samples would be performed within an hour after leaf sampling. Interestingly, Zhang et al. (2020) also provided proof-of-concept of a fast and easy-to-use point-of-care method for detection of the fungal pathogen *Magnaporthe oryzae* in rice samples. For the DNA extraction from plant samples, a filter dipstick was used after grinding of the tissue, and for detection an instrument-free lateral flow device was used. To increase sensitivity, an RPA-preamplification of target genes was included, allowing detection of DNA in the picomolar range. Although the authors indicate that sensitivity could still be improved, the test was able to clearly detect the pathogen in almost all samples tested within ~35 minutes. For other examples, we refer the reader to the following recent reviews (Sharma et al., 2021; Karmakar et al., 2022).

## 6 Nucleic acid sequencing methods

DNA sequencing has emerged as a useful tool for the identification of microorganisms (Reller et al., 2007; Barghouthi, 2011; Beye et al., 2017). By sequencing specific genetic markers and comparing the resulting sequence(s) to a reference database, the identity of a microorganism can be determined (Barghouthi, 2011). It is a more accurate and reproducible method to identify microorganisms compared to conventional techniques such as morphological and phenotypical tests (Reller et al., 2007; Tewari et al., 2011). Its use in detection and identification has accelerated together with the huge evolution in sequencing technologies in the past 15 years. While first generation sequencing technologies, with Sanger sequencing being the most popular, generate relatively long reads of up 1000 bp but is limited in throughput capacity, second generation sequencers (e.g., Illumina and IonTorrent) generate reads that are relatively short (100-300bp) but with an enormous throughput. Third generation sequencers, such as Nanopore or PacBio sequencing, are characterized by their ability to sequence single molecules and generate ultra-long reads in a high throughput manner. PacBio sequencing provides higher accuracy, but this platform demands a large initial investment and requires a lab environment. Nanopore sequencing is highly promising in the context of plant pathogen detection, as for instance the MinION platform is a relatively low cost and portable system (Loit et al., 2019). However, the sequencing accuracy is inferior to that of the PacBio sequencer and previous generations of sequencers. A detailed comparison of the methodology and technical specifications of these sequencing technologies falls outside the scope of this review, but we can refer the reader to another review paper (Hu et al., 2021). Sanger sequencing is more suitable and cost-effective for the identity confirmation of specific isolates after (semi-)selective cultivation, as mentioned above (section 3). With the advent of high-throughput 2^nd^ and 3^rd^ generation sequencers, the sequencing cost reduced considerably, which facilitated their use for multiplex detection of plant pathogens present in a sample. In addition, high-throughput sequencing can also provide information on the microbial community composition and allows detection of non-culturable organisms. Two main approaches making use of next-generation sequencing for multiplex detection of pathogens can be employed, i.e. metagenome sequencing and amplicon sequencing, which are discussed in more detail below.

### 6.1 Metagenomics

Metagenomics involves the use of shotgun sequencing of all DNA present in a sample (Quince et al., 2017). Basically, all DNA in a specific sample is extracted and sheared into smaller pieces that are massively sequenced in parallel (Sharpton, 2014). This results in a large number of sequencing reads, that are assembled into a metagenome consisting of contiguous sequences (contigs) through sequence overlap. Next, the reconstructed metagenome of a specific sample can be used to extract informative regions, that either enable identification of the microorganisms present in the sample or give more insight into the functional genes of the microbes (Quince et al., 2017; Lapidus and Korobeynikov, 2021; Semenov, 2021). However, assembly-based analysis methods have some caveats when analyzing metagenome sequencing data. Assembly of the large number of sequencing reads requires considerable computational power and complex data analysis workflows. One of the difficulties lies in differentiating highly similar genomes of closely related species, as this complicates finding the sequence overlap during the assembly process. Moreover, low abundant species often do not have sufficient sequencing coverage to generate large contigs (Quince et al., 2017; Lapidus and Korobeynikov, 2021). In such cases, assembly-free analysis methods can be used as a valuable alternative. In this approach, the obtained individual sequencing reads are directly compared to a reference genome database (Quince et al., 2017; Lind and Pollard, 2021). The advantages of this approach are that generating complex assemblies can be avoided and analysis time is reduced. Furthermore, it reduces the problems associated with low abundance species detection, as the obtained sequences are mapped directly to reference genomes. However, downsides of this approach are the need for appropriate reference databases for the samples in question (Ayling et al., 2020) and that it usually generates more false-positive hits, as the obtained sequences of universally conserved regions could be assigned to the wrong microorganism. However, the advent of third generation sequencing, also referred to as long-read sequencing, solved a variety of problems encountered in metagenomics when using second generation sequencers (Amarasinghe et al., 2020). Due to the short read lengths of the second-generation sequencing platforms, *de novo* assembly techniques are unable to resolve large repetitive regions, resulting in a highly fragmented genome assembly. In addition, several DNA regions of closely related species can be highly similar, further complicating the assembly process. In contrast, the long read lengths of third generation sequencers are able to bridge these large repetitive regions, facilitating the assembly process.

Metagenome sequencing has several advantages, including the fact that: (i) the huge amount of information may enable identification up to strain level; (ii) it allows for simultaneous and PCR bias-free detection of fungal, oomycete and prokaryotic strains; (iii) little to no *a priori* genetic information about the pathogen causing the plant disease is required (Quince et al., 2017; Sekse et al., 2017; Semenov, 2021; Aragona et al., 2022); and (iv) the technique allows for the recovery of genomes from yet uncultured microorganisms (Duan et al., 2009; Piombo et al., 2021). Especially for detection of viral plant pathogens, metagenomic approaches are useful, as viruses do not have universal genes that are targeted in amplicon sequencing, and as mentioned above, the metagenomic approach doesn’t require prior knowledge, enabling detection of plant pathogens that are yet unknown (Adams et al., 2009; Roossinck et al., 2015; Adams and Fox, 2016). Despite these advantages, the regular use of shotgun metagenomics for plant pathogen detection is still not widespread. This can be ascribed to four main reasons. First, due to the required large sequencing depth to accurately identify the microorganisms present in the sample, the technique is very expensive compared to other techniques. Second, the presence of contaminating or uninformative sequences, such as plant host DNA, can have a negative impact on the informative DNA sequences that are obtained. Third, low abundant species are difficult to detect, resulting in a lower sensitivity to detect particular pathogens as compared to targeted amplicon sequencing. And finally, the availability of suitable reference genomes for detection purposes is still limited, especially in the context of plant pathogen detection (Duan et al., 2009; Sharpton, 2014; Quince et al., 2017; Piombo et al., 2021).

Nevertheless, several examples that illustrate the value of metagenomics for detection of plant pathogens have been reported (Piombo et al., 2021). For instance, an Illumina-based metagenome sequencing approach was used to analyze the microbiome on *Arabidopsis* leaves. A reference database of 242 marker genes allowed detection and identification of several pathogenic species of *Protomyces* and *Peronospora* (Lind and Pollard, 2021). It was also recently demonstrated that long-read sequencing is highly suited for detection of agricultural and forest fungal pathogens (Loit et al., 2019), while other studies have shown the value of metagenomics approaches for detection and identification of novel plant viruses in both wheat and maize (Redila et al., 2021; Lappe et al., 2022) or for detection of the blight pathogen *Calonectria pseudonaviculata* in boxwood samples (Yang et al., 2022). The latter study showed that both the assembly-free and assembly-based detection performed well, with taxonomic identification approaching the strain level. This is in agreement with another recent study that investigated disease outbreaks of *Xylella fastidiosa* (Johnson et al., 2022), in which pathogen detection was achieved with a sensitivity similar to qPCR, and this in combination with identification at subspecies level. Furthermore, a recent case study performed by Boykin et al. (2019) demonstrated the applicability of the Nanopore MinION sequencing platform for in-field applications. The researchers sampled cassava plants in Sub-Saharan Africa for the detection of viral pathogens. The assay was able to effectively detect and identify several kinds of viruses, and was able to be performed in a timespan of about 3 hours following arrival at the site. Altogether, the results from these studies indicate that metagenomics is a useful tool for early and accurate detection of plant pathogens.

### 6.2 Amplicon sequencing

Amplicon sequencing, i.e. sequencing of an amplified marker gene, is a popular technique for the characterization of the microbial community structure. Also referred to as metabarcoding, the method relies on the amplification of a specific marker gene (DNA barcode) that is common for the target populations. The identity of the organisms is determined by comparing the DNA sequence of the amplified marker genes with a suitable reference database. The marker gene should possess the following properties: (i) it should be found in all targeted microorganisms; (ii) it should have strongly conserved regions in order to design universal primers for PCR amplification; and (iii) these highly conserved regions should encapsulate variable regions, that serve as a signature to differentiate the microorganisms (Reller et al., 2007; Hugerth and Andersson, 2017; Bush et al., 2019). The most commonly used marker gene for determination of bacterial populations is the 16S ribosomal RNA gene, while for fungi the internal transcribed spacer (ITS) in the ribosomal RNA is commonly used (Abdelfattah et al., 2018; Piombo et al., 2021; Semenov, 2021). The identity of the organisms is determined by comparing the DNA sequence of the amplified marker genes with a suitable reference database. However, 16S rRNA and ITS usually don’t provide sufficient resolution to distinguish closely related strains. In this case, sequencing of additional marker genes is required to increase the taxonomic resolution.

Amplicon sequencing has several advantages. First, compared to metagenome sequencing, the sequencing capacity is only used to determine the DNA sequence of marker genes for identification purposes, and not for sequencing plant DNA or uninformative parts of the microbial genomes, making the technique more cost-effective (Sharpton, 2014; Piombo et al., 2021). Second, there is also an abundance of established data analysis pipelines, providing a relatively user-friendly interface (Vasar et al., 2021). Third, a large number of reference sequences are available for identification purposes (Abdelfattah et al., 2018). And finally, compared to metagenome sequencing, this approach is more sensitive because of the PCR amplification step, which enables detection of low abundant species or analysis of samples with a low biomass (microbial load) (Sekse et al., 2017). The technique also has its disadvantages. Because in most cases only a short single marker gene of the target organisms is amplified, limited taxonomic resolution is obtained and usually identification only up to genus level (or in some cases at best up to species level) is obtained (Abdelfattah et al., 2018). This is especially important in the field of plant pathogen detection, where closely related species can be either pathogenic or non-pathogenic (Tedersoo et al., 2019). However, the use of third generation sequencers allows to sequence larger markers in comparison with e.g., Illumina, which considerably improves accurate taxonomic identification up to species and potentially even up to strain level. This was demonstrated for Nanopore and PacBio platforms, in which larger than conventional amplicons were sequenced (Benítez-Páez and Sanz, 2017; Tedersoo et al., 2018; Graf et al., 2021). Indeed, the increased size of the genetic marker resulted in more taxonomically informative regions that can be used for the identification, which allows for the identification of plant pathogens to the species level or possibly even strain level. In addition, the primers for universal amplification of the marker genes can have varying levels of affinity for different microbial taxa, which could lead to a bias in the amplicons that are generated. For instance, most primer pairs used for amplicon sequencing of fungi, are only able to amplify about 50% of the fungal populations (Piombo et al., 2021). Finally, some taxa will be preferentially amplified over others, providing a distorted view of the microbial community compositions. Other factors such as DNA extraction methods, as well as copy numbers of the target genes can have an influence (Brooks et al., 2015).

The value of amplicon sequencing for detection of plant pathogens has been illustrated in several cases (Piombo et al., 2021). For instance, an amplicon sequencing approach revealed that internationally traded plants often contain pathogenic oomycetes (Rossmann et al., 2021). The study was based on the use of the ITS1 region targeting fungal microorganisms. However, the identification could only be reliably performed up to the genus level. Species level identification was not possible, due to the short reads of the Illumina platform. Another example involves detection of fungi in soil samples by amplicon sequencing of the full length ITS region with PacBio sequencing. Due to the longer read lengths, this platform achieved species-level resolution of the fungi present in the sample (Tedersoo et al., 2018). A similar study used nanopore for amplicon sequencing of a *Xylella-*specific marker gene to detect and identify *Xylella* spp. in leaves. Again, longer read lengths obtained by a third generation sequencing platform allowed a subspecies level resolution, moreover, the results could be obtained in as fast as 15 minutes of sequencing (Marcolungo et al., 2022). An amplicon sequencing approach using conventional MLSA marker genes even allowed identification to strain level for *Xylella fastidiosa* (Faino et al., 2021).

## 7 Biosensors

Biosensors comprise devices that consist of a biorecognition element combined with a physicochemical transducer that generates a measurable signal upon the binding of the target analyte with the biorecognition element (Hameed et al., 2018; Bridle and Desmulliez, 2021). Biosensors are promising tools for point-of-care applications, as they are generally low-cost, easy to use, and can provide fast results (Bridle and Desmulliez, 2021). Common examples of transducers include: (i) electrochemical transducers, which detect the binding event based on changes in voltage, impedance, or conductance; (ii) mass-based transducers, which detect a resonance frequency change based on mass change when the target analyte binds to the biorecognition element; and (iii) optical transducers that detect differences in the reflection of incoming light upon binding of a target analyte to the biorecognition element (Fang and Ramasamy, 2015; Hameed et al., 2018; Bridle and Desmulliez, 2021). For an in-depth discussion on biosensors suitable for plant pathogen detection we refer to the review of Buja et al. (2021).

Several types of biorecognition elements can be used, but most types make use of nucleic acid probes, antibodies, aptamers, or enzymes to detect a target analyte. The use of antibodies and nucleic acid probes to detect a target analyte is already explained in section 4 and 5. Aptamers are also highly suited for implementation in biosensors because they are easily labeled, and they show a conformational change upon binding of the target analyte (Toh et al., 2015; Khater et al., 2017; Shahdordizadeh et al., 2017; Bridle and Desmulliez, 2021). The use of enzymes is based on the conversion of a specific target analyte with high specificity and high affinity. However, the enzyme-based approach is mostly used for detection of specific substrates and is less suited for pathogen detection. The choice of the biorecognition element depends on the type of transducer that is used, and on the type of molecule that is targeted. While aptamers and antibodies target a specific antigen, nucleic acid probes are used to target a specific DNA sequence. In addition, the ease of labelling, the ease of immobilization, and the type of sample that is analyzed will play a role in selecting the most suited biorecognition element, as all mentioned factors influence the final cost, robustness, sensitivity, etc. of the biosensor. The immobilization of the biorecognition elements on the sensing surface plays a big role in the efficacy of the biosensors (Cardoso et al., 2022). The goal of immobilization is to fix the biorecognition element to the electrode, and to assure an optimal packing density and orientation of the recognition elements (Cesewski and Johnson, 2020). Different immobilization methods exist such as adsorption-based techniques, covalent attachment, avidin and biotin systems as well as self-assembled monolayers.

**Figure 6:**
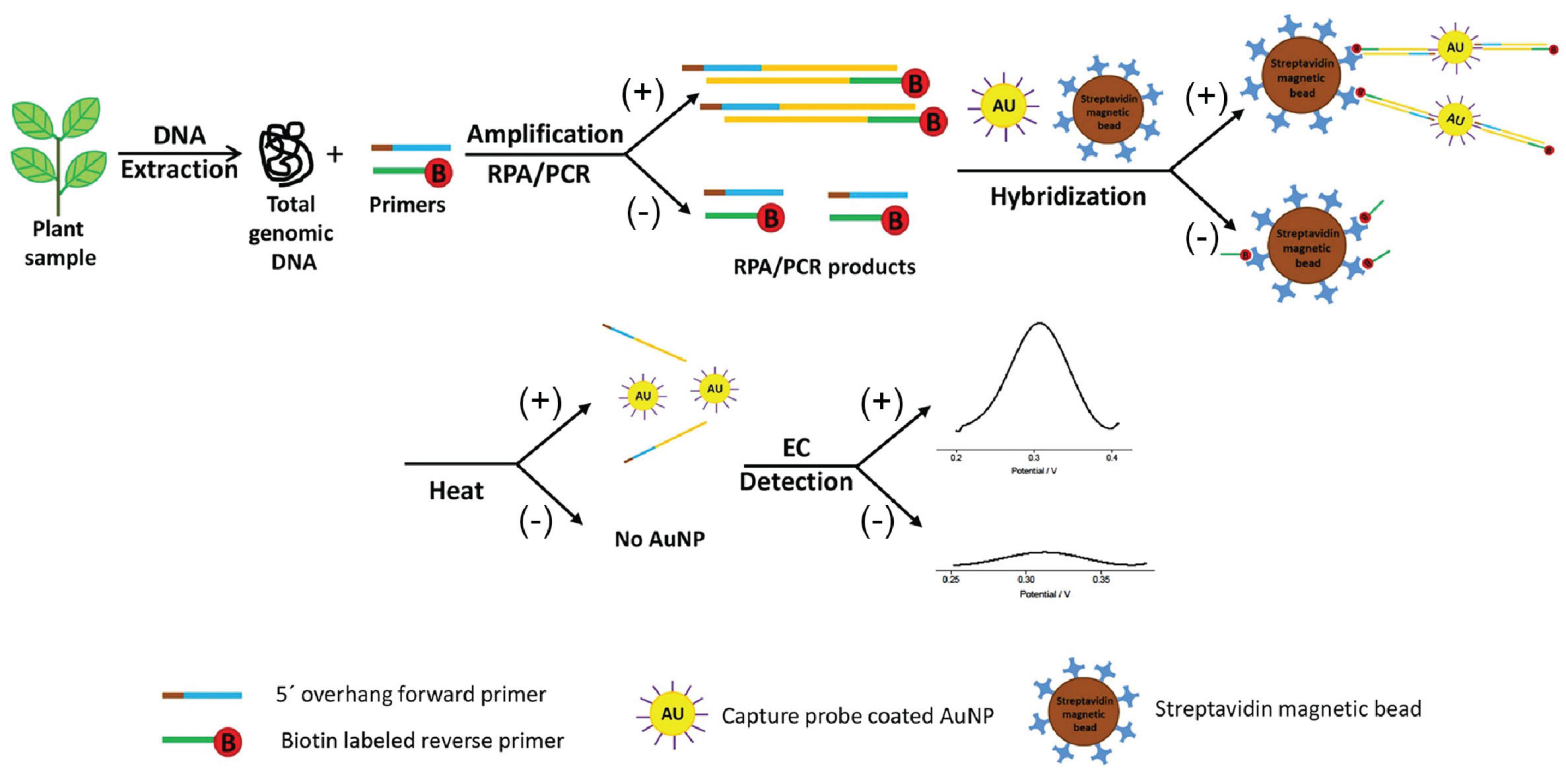
Schematic presentation of the working principle of a biosensor based on RPA amplification to detect plant pathogens. The DNA extracted from a(n) (infected) plant sample is subjected to RPA amplification. The reverse primer is labelled with biotin, while the forward primer contains a 5’ addition complementary to oligonucleotide probes that are bound to gold particles. Should the sample contain the target pathogen (indicated by (+) in the figure), amplification will occur, resulting in amplicons labeled with a biotin label on one end and a DNA sequence complementary to the probes on the gold particles on the other end. The amplified product is incubated together with streptavidin magnetic beads and gold nanoparticles coated with capture probes, and will form a complex if amplification of the target sequence occurred. If the sample contains no target DNA (indicated by(-)), no labeled amplicons are formed. After magnetic separation of the complex, the complex is dissociated by heat treatment, resulting in the release of the gold nanoparticles. The gold nanoparticles will be deposited on an electrode surface, and through differential pulse voltammetry a characteristic signal is obtained, which indicates the presence of a pathogen (adapted from Lau et al., 2017).

The wide variety of different transducers and biorecognition elements enables the development of a wide range of different types of biosensors that can be used for detection of plant pathogens, as illustrated in the following examples. A first example involves a DNA hybridization-based biosensor for detection of *Phytophthora ramorum* in rhododendron leaves (Yüksel et al., 2015). Gold nanoparticles were coated with nucleotide capture probes specific for the *ypt1* gene of *P. ramorum*. Hybridization of the target amplicon to the probes was detected with surface enhanced raman spectroscopy. No details were given regarding the sensitivity of the technique, but no cross-reactivity with the closely related *P. lateralis* species was observed. In another study, an electrochemical biosensor based on RPA amplification was developed for detection of *Pseudomonas syringae* (Lau et al., 2017). After DNA extraction of the sample, an RPA was performed with the reverse primer labelled with biotin, and the forward primer containing a 5’ addition complementary to oligonucleotide probes that are bound to gold particles (**Figure 6**). Amplification products were incubated with magnetic beads coupled with streptavidin (to bind the biotin-labeled reverse primer) and gold nanoparticles coupled with oligonucleotides to hybridize with the forward primer. Application of a magnetic field allows to separate the magnetic beads (bound with either the reverse primer only or with the amplified product). Next, the products are heat-treated to release the gold nanoparticles, which are used as a label to coat the detection probe. The gold nanoparticles are detected through differential pulse voltammetry, and the corresponding signal was proportional to the amount of gold nanoparticles, which is a measure for the amount of pathogen in the sample. The assay was approximately 10 000 times more sensitive than a conventional PCR assay. The large increase in sensitivity can be ascribed to two factors: (i) RPA can amplify a lower amount of DNA; and (ii) the electrochemical biosensor was 100 times more sensitive than visualization on a gel. Furthermore, the assay can be performed within 60 minutes. Another example involves the use of an immunoassay biosensor for detection of *Citrus tristeza* virus in infected citrus samples (Freitas et al., 2019). The assay consists of a probe, which is surface-coated with capture antibodies, and a magnetic bead coated with a secondary antibody that is conjugated to an HRP enzyme. The primary antibody-antigen-secondary antibody complex is isolated by making use of the magnetic beads coupled to the secondary antibody. Presence of the target antigen is detected by the amperometry-based biosensor that senses the redox reaction caused by the HRP enzyme. The limit of detection of this biosensor was 0.3 fg/ml, and the assay could be performed in 50 minutes. The cost was $1.99 (US) whilst a comparable ELISA assay cost $8.30 per microwell. A last example involves a non-invasive volatile organic compound (VOC) biosensor, integrated on a smartphone, for detection of late blight in tomato leaves (Li et al., 2019). When infected with *P. infestans*, a plant will emit certain VOC markers. These markers can be detected by cysteine-functionalized gold nanoparticles, which upon exposure to certain VOCs cause them to aggregate, leading to color formation. The cysteine-functionalized nanoparticles can be applied to a disposable paper strip, which can be inserted into the smartphone-based device. This device is linked with a small battery-powered pump, which can draw air from the sample environment over the paper strip, causing the nanoparticles to aggregate in the presence of VOCs indicative for a specific disease. The resulting colorimetric change can then be analyzed by the smartphone camera and used to provide accurate and early detection of late blight in tomato leaves, as well as other disease-related plant VOCs. The device has been validated through blind testing using both artificially inoculated tomato leaves and field-collected infected leaves.

## 8 Discussion

Plant pathogens are responsible for an up to 40% yield loss of economically important crops each year. In order to reduce yield losses, it is of utmost importance to detect pathogens as early as possible, and preferably even before disease symptoms are visible. The importance of having a suitable detection and identification technique cannot be stressed enough, as it allows for efficient and early disease remediation strategies to be undertaken. In that regard, good detection techniques are an important pillar in integrated pest management, which aims to reduce the use of chemical pesticides to an absolute minimum, thereby contributing to a more sustainable agriculture. However, although routine sampling can be used for disease detection, the spatial variation of pathogens inside the plant itself (e.g., leaf, stem, roots) as well as in fields can make preventative monitoring for latent infections tedious, time-consuming, and labor-intensive. The use of remote sensing technologies could provide a possible solution in this matter, as they allow for localization of areas in the field where plants are exhibiting stress, even before they show visible disease symptoms.

However, diagnostic tools to detect plant pathogens have applications that go well beyond their use in the field to monitor plant diseases. Due to the growing global trade, the risk of spreading plant pathogens has considerably increased. This requires adequate monitoring of import and export products, and if necessary, implementing the proper phytosanitary measures (PM). Import or export of plants therefore often requires plant passports or phytosanitary certificates, which guarantee that the plants are pathogen-free, as for example stipulated by EU regulation (Regulation (EU), 2017/625) (Buja et al., 2021). To aid National Plant Protection Organizations (NPPO) in their control of plant pests and diseases, the EPPO published standards with guidelines and recommendations on monitoring of plant pests and diseases, and corresponding phytosanitary measures. For instance, EPPO provides a comprehensive overview of specific diagnostic protocols (*EPPO Standards - PM7 Diagnostic Protocols for Regulated Pests*) that have be applied to monitor the presence of a dedicated list of plant pathogens and quarantine organisms (*EPPO Standards - PM 1 General Phytosanitary Measures*). This means that for each pathogen, a number of validated assays (e.g., plate count methods, bioassays, serological and molecular tests) are recommended for detection in (a)symptomatic plants. Although these techniques are currently the golden standard and recommended by the authority, they have their drawbacks, including the need for trained personnel, high costs, and in some cases have an especially long processing time that leads to a (too) late detection (Buja et al., 2021). This clearly points to the need for development of detection techniques that are fast, sensitive, allow accurate identification and quantification of the pathogen, are able to detect multiple pathogens in a single test (multiplexing), are low-cost, and can be used at point-of-care. Such techniques are especially useful when increased monitoring is demanded by the government (Buja et al., 2021). The main aim of this review was therefore to present an overview of methods that are currently available to detect plant pathogens, and discuss their main advantages and disadvantages (**Table 2**).

**Table 2:** Overview of the relevant specifications of the different detection methods discussed in this study.

	Specificity^4^	Sensitivity^4^	Ease of use^4^	Analysis time^4^	Throughput^4^	Multiplex capacity^5^	Quantification^5^	Point of care^5^	Cost^6^
**Cultivation-based method**	*	***	***	*	*	N	Y/N	N	€
**Immunoassays^1^**									
** ELISA**	**	*	***	***	***	N	Y	N	€€
** LFIA**	**	*	****	****	*	N	N	Y	€
**PCR^2^**									
** cPCR**	**	**	**	**	*	N	N	N	€€
** mPCR**	**	*(*)	**	**	*	Y	N	N	€€
** qPCR**	***	***	**	***	***	Y/N	Y	N	€€
** ddPCR**	***	****	*	**	**	Y/N	Y	N	€€
**Isothermal amplification^3^**									
** LAMP**	***	**	***	****	*	N	N	Y	€
** RPA**	**	**	***	****	*	Y/N	Y/N	Y	€
**Hybridization**									
** Macroarrays**	***	**	*	*	*	Y	Y/N	N	€€€
** CRISPR-Cas**	***(*)	***(*)	**	***(*)	*	Y/N	Y/N	Y	€
**Sequencing**									
** Metagenomics**	****	**	*	*(*)	****	Y	Y/N	N	€€€€
** Amplicon sequencing**	***	***	*	*(*)	****	Y	Y/N	N	€
**Biosensors**	**(*)	**(*)	***	****	*	Y/N	Y	Y	€

^1^ ELISA, enzyme-linked immunosorbent assay; LFIA, lateral flow immunoassay.

^2^ cPCR, conventional PCR; mPCR, multiplex PCR; qPCR, quantitative PCR; ddPCR, digital droplet PCR.

^3^ LAMP, loop-mediated isothermal amplification; RPA, recombinase polymerase amplification.

^4^ The number of asterisks is a measure for the performance of the techniques with regard to each property (*moderate; **good; ***very good; ****excellent). An asterisk between brackets (*) indicates that depending on which approach is taken, the performance in that aspect is increased.

^5^ For each technique it is indicated if multiplexing, quantification, or use at point-of-care is possible (Y), limited (Y/N) or not (N).

^6^ The number of € symbols is a measure for the analysis cost, based on a survey of routine laboratories: € (0-50 EUR), €€ (50-100 EUR), €€€ (100-150 EUR), and €€€€ (+150 EUR).

While cultivation-based techniques are still valuable because of their simplicity and low cost, they have severe limitations regarding specificity, sensitivity, and analysis time. In contrast, immunological assays are characterized by a high specificity, fast analysis time, limited sample preparation, and can be performed within a couple of hours. However, immunological assays usually have a low sensitivity. PCR-based approaches do combine a high sensitivity with high specificity and fast analysis time, but require more sample preparation time for DNA/RNA extraction and is preferably done in a lab environment. Isothermal amplification techniques can circumvent this, and are perfectly suited for in-field detection because they require limited or no sample preparation, a simple heat block, and a user-friendly interpretation of the results. Combining isothermal nucleic acid amplification techniques with easy interpretation procedures, e.g., by using a lateral flow device, allows for a fast interpretation of the amplification results. However, conventional PCR and isothermal amplification often lack the capacity to quantify pathogens, in contrast to qPCR-methods. Recent developments in mentioned techniques, include increased automation, ease-of-use, and miniaturization, as for instance shown in the use of biosensors.

Nevertheless, the main limitation that is common for the abovementioned techniques is their limited capacity of multiplex detection. Although in some cases (e.g., multiplex PCR), a few pathogens can be detected simultaneously, in most cases a specifically developed and validated assay has to be used to detect each individual target pathogen. Considering that many different pathogens can form a threat on crops, multiple tests are needed to exclude the presence of plant pathogens. Hybridization-based techniques could resolve the issue of the limitations in multiplexing. Micro- or macroarrays make use of a range of detector probes for the detection of multiple pathogens in a single test. However, the higher cost and limited commercial availability hampers the widespread adoption of micro- or macroarrays and it still requires *a priori* genetic information of the target pathogens. A promising alternative for multiplex detection lies in next generation sequencing techniques, either through the use of amplicon sequencing or *via* metagenomics. These techniques do not require *a priori* knowledge of the plant pathogen. On the contrary, it can provide genetic information of all microorganisms present in a sample, including yet unidentified pathogens. However, routine use of amplicon or metagenome sequencing is still limited due to the relatively high costs. Most promising in this regard seem to be the third generation sequencers, whose long reads provide better identification potential compared to the short reads of the second generation sequencers.

Although several new technologies for plant pathogen detection have emerged the last decade, it is crucial that these are thoroughly validated regarding specificity and sensitivity, not only with pure cultures or pure DNA samples, but also with plant samples spiked with the target pathogen. Furthermore, each new technique should be benchmarked with more conventional methods and should also be cost-effective before their use in practice (Cardwell et al., 2018).

In conclusion, it is clear that the ideal detection method is not yet available, and the choice of which detection method should be used is widely dependent on the target pathogen, the available budget, the sample matrix, as well as the technological availability of that area. However, continuous efforts are made to develop new technologies that are increasingly adopted in modern plant disease monitoring.

## Author contributions

MV and HR conceived the concept and structure of the review. The initial draft was written by MV and HR. SC did market search on analysis costs. All authors contributed to writing. Final revision was done by MV and HR. All authors contributed to the article and approved the submitted version.
